# Inverted azolophanes: alternant *o*-heteroarene/*p*-arene macrocycles

**DOI:** 10.1039/d5sc05981j

**Published:** 2025-09-23

**Authors:** Yun-Hsien Lin, Xiqu Wang, Dariusz W. Szczepanik, Paweł A. Wieczorkiewicz, Ognjen Š. Miljanić

**Affiliations:** a Department of Chemistry, University of Houston 3585 Cullen Boulevard #112 Houston TX 77204-5003 USA miljanic@uh.edu; b K. Gumiński Department of Theoretical Chemistry, Faculty of Chemistry, Jagiellonian University Gronostajowa 2 30-387 Kraków Poland dariusz.szczepanik@uj.edu.pl; c Faculty of Chemistry, Warsaw University of Technology Noakowskiego 3 00-664 Warszawa Poland; d Faculty of Chemical Engineering, Industrial University of Ho Chi Minh City Ho Chi Minh City 71408 Vietnam

## Abstract

Using the Debus–Radziszewski reaction, eight imidazole-based macrocycles were synthesized from cyclotetrabenzil, while three oxazole analogs were prepared by the Davidson oxazole synthesis starting with cyclotetrabenzoin esters. These macrocycles were dubbed “inverted azolophanes” as their azole valences point divergently outside of the central ring, in contrast to the more studied azolophane architectures. Crystal structures of five macrocycles were obtained and show largely coplanar fusion of the 24-membered central macrocycle and the four azole rings. Despite the formal possibility of a 24-membered antiaromatic ring current, inverted azolophanes show ring currents firmly localized in their six- and five-membered rings. The roughly square-shaped connectivity of the available azole valences and the shape-persistent nature of the macrocycles bode well for their use as tetragonal building blocks for the construction of ordered frameworks.

## Abstract 2

Using the Debus–Radziszewski reaction, eight imidazole-based macrocycles were synthesized from cyclotetrabenzil, while three oxazole analogs were prepared by the Davidson oxazole synthesis starting with cyclotetrabenzoin esters.

## Graphical abstract

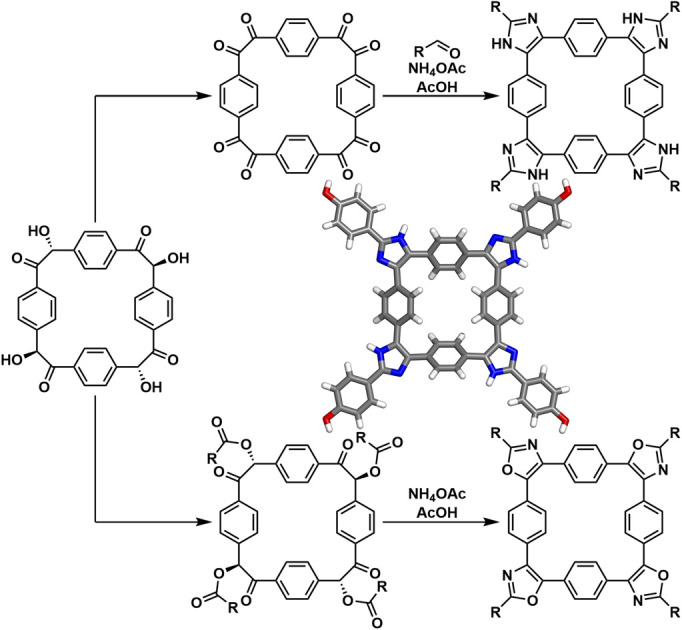

## Introduction

**Fig. 1:**
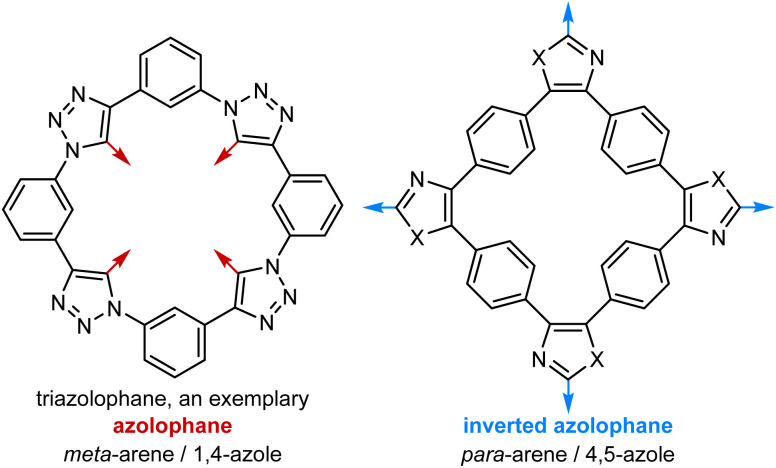
Exemplary structures of an azolophane (left) and an inverted azolophane (this work, right).

Research into two-dimensional materials has blossomed since the discovery of graphene^1^ and the extension of the exfoliation strategy to covalent-organic frameworks^2^ and synthetic two-dimensional polymers.^3^ Planar or nearly planar small molecules hold an important place in these studies as they serve as soluble and thus readily characterized models for the behaviours of polymeric systems. Among the flat macrocycles with alternating homo- and heteroaromatic motifs, the class of azolophanes^4^ (Fig. 1, left) has been particularly popularized by Flood *et al.* and their work on anion binding *via* convergent [C–H···anion] interactions.^5^ In this contribution, we report the preparation and characterization of “inverted azolophanes” (Fig. 1, right): cyclobenzoin-derived macrocycles in which *p*-phenylene rings alternate with *o*-imidazolylene or *o*-oxazolylene rings. Because of their 4,5-connectivity on the heterocycle and the limited space within the macrocycles, such azolophanes are forced to invert and orient their C–H/C–R azole valences away from the central macrocyclic core.

The cyclobenzoin^6^ family of macrocycles is readily synthesized and has expanded to include cyclobenzoin esters,^7^ oxidized cyclobenzil diketone derivatives,^8^ and their condensation products.^9^ These diverse but related molecular architectures have been used as supramolecular hosts for linear guests,^7^ iodine capture platforms,^9*a*^ components of organic batteries,^8,10^ and precursors to porous organic polymers (POPs).^11^

## Results and discussion

**Scheme 1:**
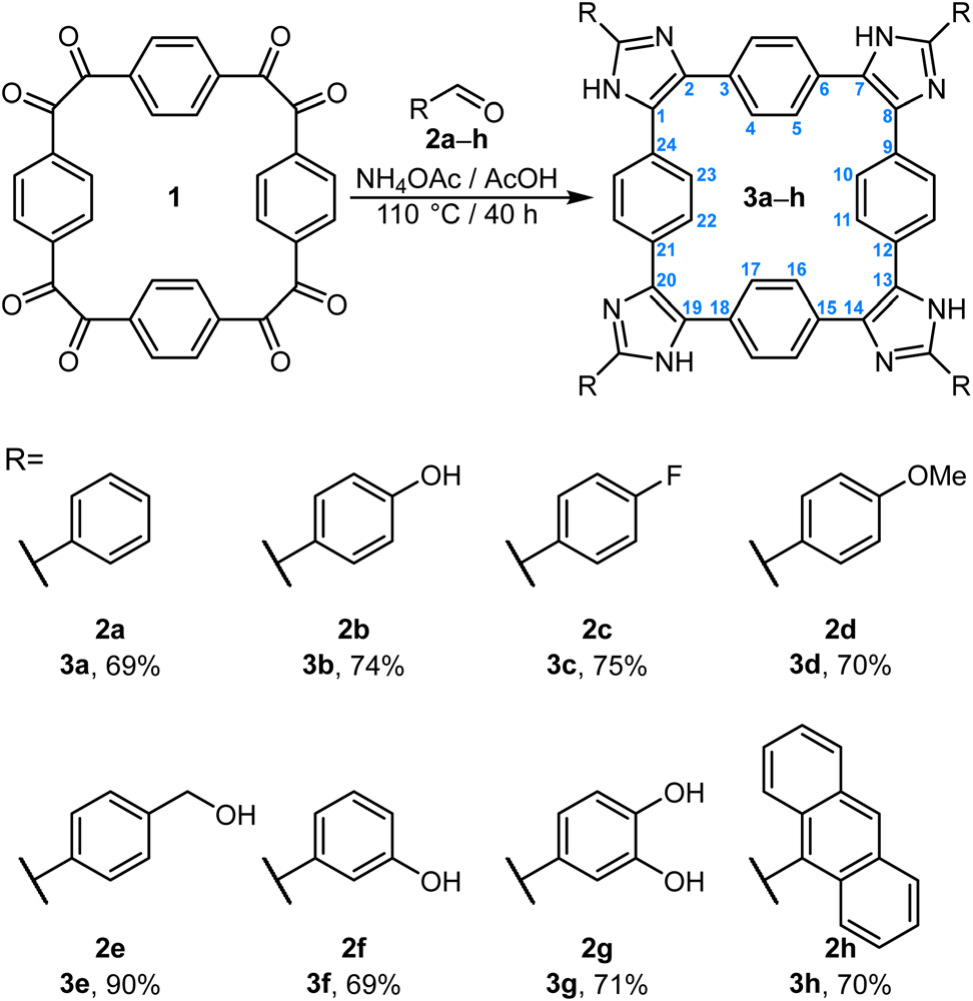
Synthesis of the imidazole-based macrocycles 3a–h. Shown in blue is the numbering scheme that is used in the text.

**Scheme 2:**
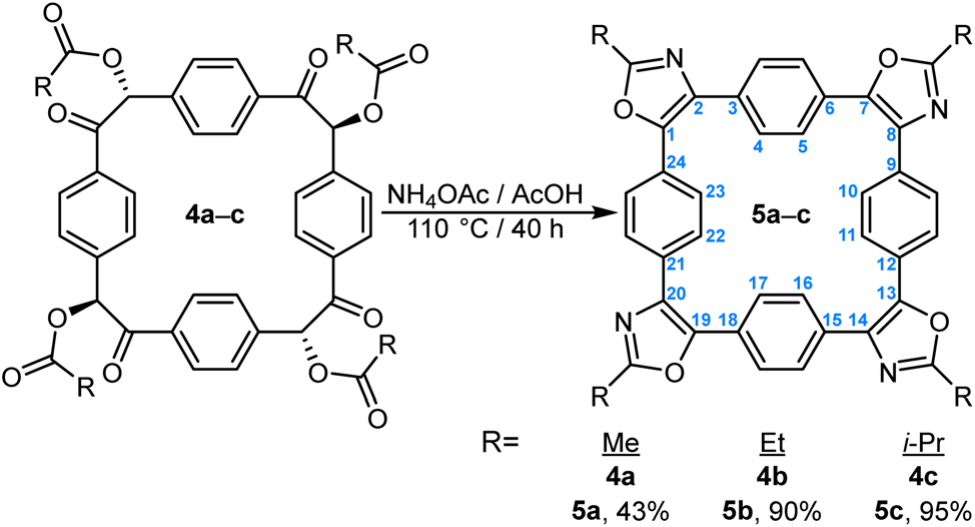
Synthesis of the oxazole-based macrocycles 5a–c. Shown in blue is the numbering scheme that is used in the text.

Imidazole-based macrocycles 3a–h (Scheme 1) were synthesized from cyclotetrabenzil (1)^9*b*^ in yields ranging from 69 to 90% using the Debus–Radziszewski reaction.^12,13^ In an analogous procedure, oxazole-based macrocycles 5a–c (Scheme 2) were prepared in 43–95% yields from cyclotetrabenzoin esters 4a–c (ref. 7b) using the Davidson oxazole synthesis.

To establish whether the formation of 3a–h is reversible, we exposed macrocycle 3b to aldehyde 2f under the original reaction conditions shown in Scheme 1. After 40 h at 110 °C, only the starting materials were isolated, without any evidence of the formation of 3f; this observation confirmed that, once aromatized, 3a–h do not revert to 1 or any of the intermediates.

Compounds 3a–h and 5a–c are powders ranging in color from yellow to almost black, and with fluorescence in the solid state and solution (*vide infra*). Their ^1^H and ^13^C NMR spectra are consistent with their structures, albeit complicated by signal broadening which is a consequence of the hindered rotation of the phenylene rings around their central axes and/or the imidazole tautomerization in 3a–h which is slow on the NMR time scale. In some cases, their ^1^H NMR spectra, taken in DMSO-*d*_6_ at 60–100 °C, showed sharpening of the peaks, accompanied by some decomposition (and presumably decomposition of DMSO-*d*_6_ as well).

**Fig. 2:**
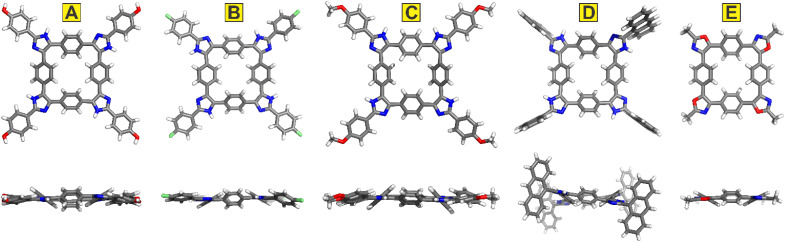
Top (top row) and side (bottom row) views of crystal structures of 3b (A), 3c (B), 3d (C), 3h (D), and 5a (E). Element colours: C—grey, H—white, O—red, N—blue, F—lime green. Solvent molecules and disorder omitted for clarity.

We obtained diffraction-quality single crystals of several prepared macrocycles. Crystals of imidazole 3b were grown by slow vapor diffusion of *n*-pentane into its solution in 1,4-dioxane and *N*,*N*-diethylformamide (DEF); those of 3c by slow diffusion of MeOH vapors into its solution in DEF; those of 3d by vapor diffusion of MeOH into its solution in 2-methoxyethanol; finally, crystals of 3h were grown by slow diffusion of *n*-pentane into its solution in dioxane. Diffraction-quality crystals of oxazole 5a were produced by slow diffusion of Et_2_O into the solution of 5a in 1,2-dichloroethane. Their crystal structures are shown in Fig. 2 alongside one another.

Macrocycle 3b crystallizes in the *P*2/*n* space group with four molecules of 3b, twelve molecules of DEF, and four molecules of dioxane per unit cell. The macrocyclic skeleton and the four imidazole rings are effectively coplanar (Fig. 2A), with the phenylene rings of 3b rotated with respect to the average plane of the macrocycle by 30.9, 30.9, 34.4, and 36.7°.

Imidazole 3c crystallizes in the *P*1̄ space group with two molecules of 3c, two molecules of DEF, and four molecules of MeOH per unit cell. The macrocyclic skeleton and the four imidazole rings are once again effectively coplanar (Fig. 2B): excluding carbon atoms number 4, 5, 10, 11, 16, 17, 22, and 23 in Scheme 1, the greatest deviation from the average plane of the macrocycle is lower than 0.1 Å. On the other hand, the phenylene rings of 3c are rotated with respect to the average plane of the macrocycle by 26.8, 32.1, 39.0, and 40.9°.

Imidazole 3d crystallizes in the *P*2_1_/*n* space group with four molecules of 3d and twenty molecules of MeOH per unit cell. The macrocycle and the four imidazole rings are coplanar (Fig. 2C), while the phenylene rings of 3d are rotated with respect to the average plane of the macrocycle by 25.2, 27.9, 43.7, and 45.0°. The methoxyphenyl substituents are positioned at angles of 15.1–27.8° relative to the central plane of the macrocycle.

Anthracenyl-substituted 3h crystallizes in the *P*1̄ space group with two molecules of 3h and two molecules of dioxane in the unit cell. Some disorder is evident in the anthracene rings. The central macrocycle is twisted from planarity (Fig. 2D, bottom) in a saddle-like fashion, with the carbon atoms at the macrocycle-imidazole fusion deviating from the plane of the central ring by 0.34–0.52 Å. The anthracene rings are roughly perpendicular to the central macrocycle, with interplanar angles of 87.8, 86.8, 84.8, and 75.6°.

Crystal structure of 5a (Fig. 2E) also reveals a nearly planar molecule, with the phenylene rings rotated slightly out from the average plane of the molecule. The greatest deviation from the average plane of 0.79 Å for one of the carbon atoms on those phenylene rings.

**Fig. 3:**
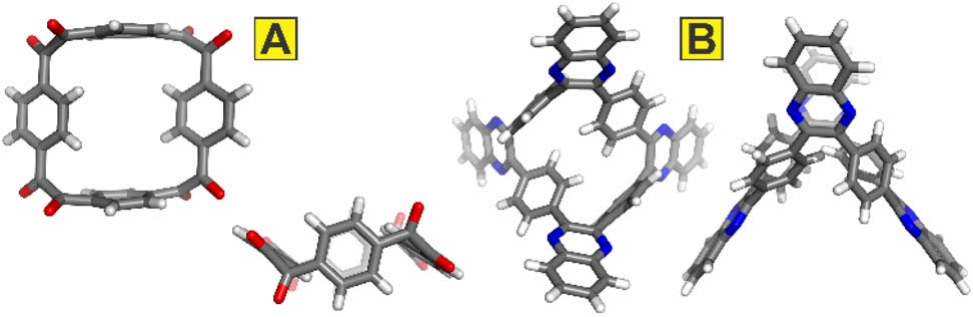
Top and side views of the crystal structures of 1 (A) and its quinoxaline derivative 6 (B) show highly deplanarized structures, in sharp contrast to 3b–d, 3h, and 5a. Element colours: C—grey, H—white, O—red, N—blue.

In all the structures, angles established between the C2 atoms of two neighboring imidazole/oxazole nuclei and the macrocycle's centroid are very close to 90°: 89.8–90.9° in 3b, 88.9–90.9° in 3c, 88.8–91.5° in 3d, 89.6–92.1° in 3h, and 89.8–90.2° in 5a. The inverted azolophanes therefore constitute a convenient 90° tetravalent building block for the construction of ordered structures, characterized by structural modularity and facile synthesis (in contrast to *e.g.*, porphyrins). Their roughly planar structures stand in sharp contrast to both 1 (Fig. 3A) and its quinoxaline derivatives such as 6 (Fig. 3B),^11*b*,*c*^ in which the six-membered ring fusion forces the macrocycles into a saddle-shaped geometry.

**Fig. 4:**
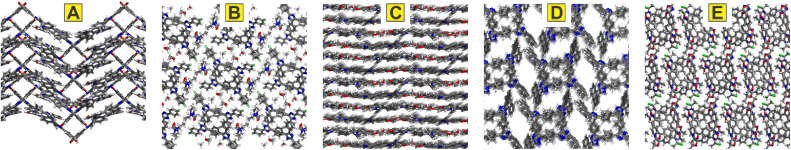
Extended crystal packing diagrams of 3b (A), 3c (B), 3d (C), 3h (D), and 5a (E). Element colours: C—grey, H—white, O—red, N—blue, F—lime green, Cl—green. Disordered solvent molecules omitted for clarity.

While the molecular structures of the crystallographically studied inverted azolophanes show a great degree of similarity, their extended packing differs significantly (Fig. 4). Imidazole 3b organizes into a complex zig–zag pattern (Fig. 4A) through the intermolecular [O–H⋯N] hydrogen bonds, as well as a multitude of solvent-mediated short contacts. Compound 3c packs into corrugated 2D sheets through [C–F⋯H–C] contacts of 2.45 Å and contacts with the intervening molecules of DEF (viewed from the top in Fig. 4B), while 3d similarly packs into 2D sheets (with MeOH solvent molecules) that stack on top of one another along the crystallographic *a* axis (viewed from the side in Fig. 4C). As could be expected, anthracene moieties dominate the crystal packing of 3h, wherein four molecules come together to establish T-shaped interactions between their anthracenyl groups with interplanar angles of 64.1, 62.6, 67.2, and 70.9° (Fig. 4D). Nestled between thus organized molecules of 3h are dioxane solvent molecules which hydrogen bond with 3h. Finally, the extended zig–zag packing of 5a (Fig. 4E) is akin to that of 3b: mediated by the short contacts established between the oxazole heteroatoms and the C–H bonds of the methyl group, as well as by the [C–H⋯X] contacts between the oxazole moieties and the included 1,2-dichloroethane solvent molecules.††Oxazoles' nitrogen and oxygen atoms are crystallographically disordered. The resultant sheets stack along the crystallographic *b* axis.

Macrocyclic structures of 3 and 5, composed of alternating *para*-linked six-membered (6MR) phenyl rings and five-membered (5MR) heterocycles, feature a conjugated bonding network that could, in principle, support global (anti)aromaticity across the 24-membered macrocyclic perimeter (24MR). To qualitatively explore this possibility, we employed the bond delocalization function (BDF)^14^ and anisotropy of the induced current density (AICD),^15^ while the quantitative analysis relied on averaged populations of cyclically delocalized π-electrons derived from the electron density of delocalized bonds (EDDB)^16^ method and the nucleus-independent chemical shifts calculated exactly 1 Å above/below the centroid of 6 MR and 5MR units, NICS(1).^17^ All calculations have been performed at the ωB97X-D/def2-TZVPP level of the density functional theory utilizing Gaussian G16.C01 software.^18–20^ Spectral MO-resolved decomposition was performed using Gaussian G16 and was invoked by adding the following keywords to the route: NMR(CSGT) IOP(10/93=2).

**Fig. 5:**
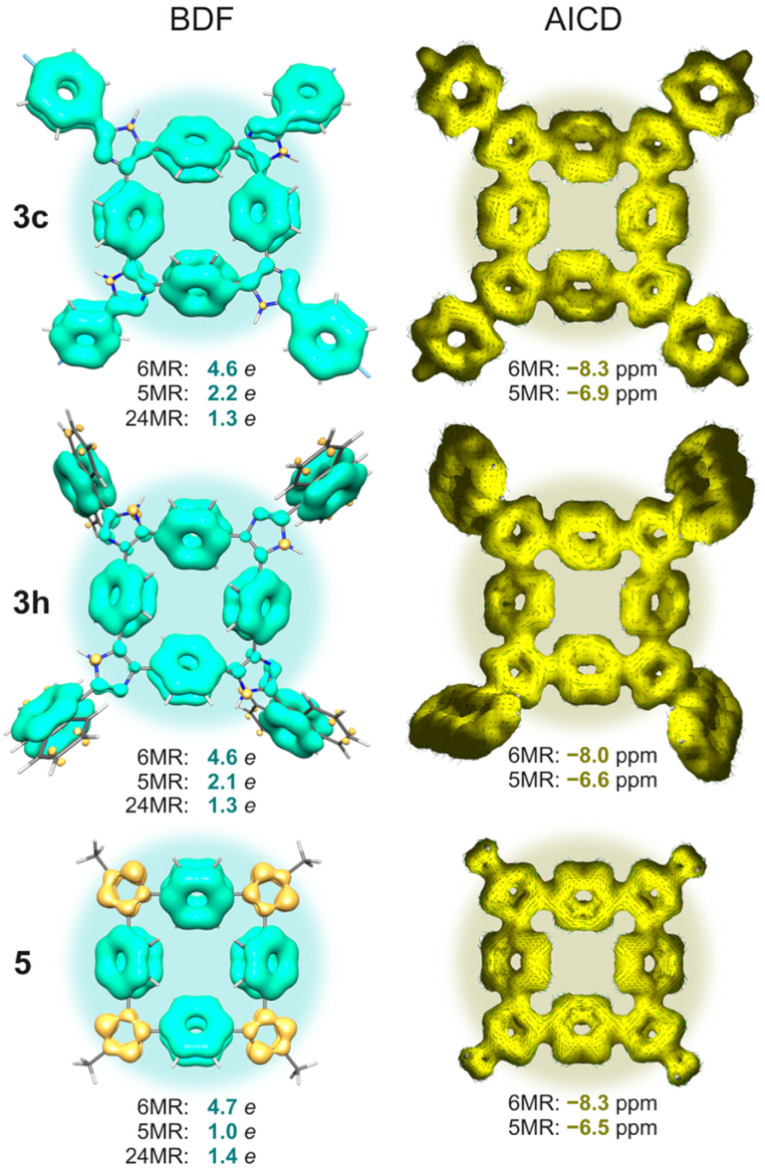
Visualization of the resonance π-bonding patterns (left, BDF) and the magnetically-induced current densities (right, AICD) for macrocycles 3c, 3h, and 5a. Positive (cyan) and negative (yellow) values of BDF correspond to delocalized (aromatic) and localized (olefinic) bonding, respectively. EDDB populations and NICS(1) values are reported for selected rings: phenyl (6 MR), heterocyclic (5MR), and the full macrocyclic perimeter (24MR).

The BDF isosurfaces for 3c, 3h, and 5a (which were chosen as exemplary structures, Fig. 5) reveal that cyclic π-delocalization is the strongest within the phenyl rings, significantly weaker in the imidazole cycles, and essentially suppressed in the oxazole rings, where the high electronegativity of oxygen atoms promotes mostly olefinic (localized) bonding character. This is consistent with both the calculated and the crystallographically determined bond lengths, showing a pronounced contraction of the bridging C–C bond to 1.36 Å in 5a. The EDDB-based electron population analysis unambiguously rules out global aromaticity of the 24MR perimeter, which accumulates only 1.3–1.4 *e* (*i.e.*, ∼0.06 *e* per atom). The π-sextets remain localized in the phenyl rings, preserving up to 85% of benzene's aromatic character (EDDB ≈ 5.5 *e*; ∼0.92 *e* per atom), while local heteroaromaticity in the 5MR units is substantially lower, ranging from 18% (1.0 *e* in 5a) up to 40% (2.2 *e* in 3c) of the benzene's value.

The AICD current maps confirm this picture, displaying local diatropic currents over both 6MR and 5MR units. NICS(1) values in the range of −8.3 to −6.5 ppm (*vs.* −10.2 ppm for isolated benzene) further support local aromatic character. Due to the size-extensivity issue of NICS,^21^ a direct comparison between 6MR and 5MR values is not meaningful. Notably, the direct *para*-linkage of alternating 6MR and 5MR units induces current interference effects, which may give the visual impression of a weak global paratropic current around the 24MR perimeter. However, this is illusory: a spectral MO-based decomposition of the NICS(1) values reveals no significant virtual HOMO → LUMO rotational transitions—a hallmark of true magnetic antiaromaticity, according to the Fowler–Steiner selection rules.^22^ Indeed, in each system, the HOMO contributes less than 0.1 ppm to the total NICS(1) signal.

To summarize, although the macrocyclic perimeter in 3c, 3h, and 5a formally satisfies Hückel's rule for antiaromaticity, BDF, EDDB, AICD, and NICS analyses reveal that cyclic delocalization of electrons remains strictly local, confined to phenyl subunits. The presence of weak paratropic current loops in AICD is merely a visual artifact arising from the interference of local diatropic currents—not a signature of global magnetic antiaromaticity.

**Fig. 6:**
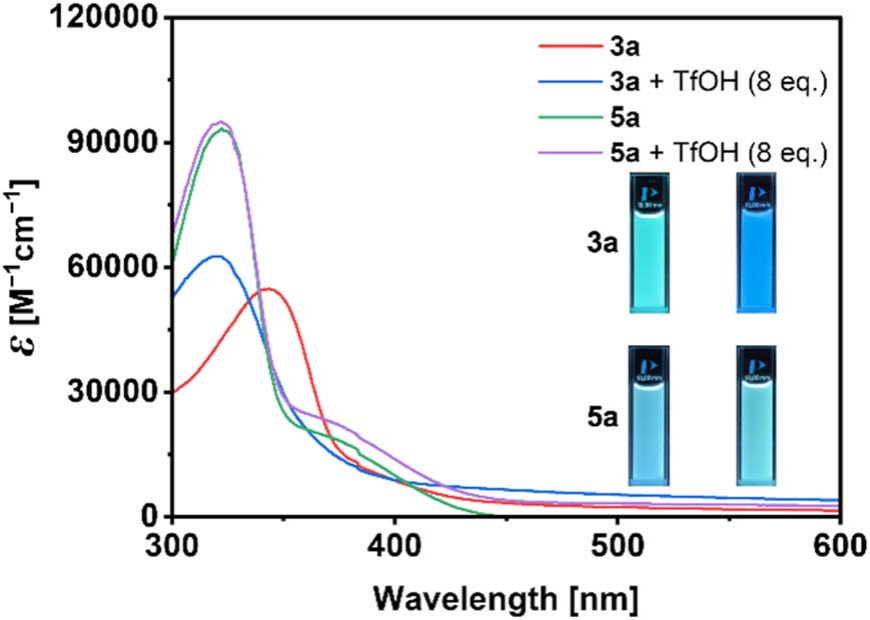
UV/vis absorption spectra of 3a (8.9 × 10^−6^ M solution in THF) and 5a (7.8 × 10^−6^ M solution in THF) before (red and green curves, respectively) and after the addition of 8 eq. of TfOH (blue and magenta curves, respectively). Inserts show the solutions' fluorescence before (left vials) and after (right vials) exposure to TfOH (*λ*_exc_ = 254 nm).

UV/vis absorption spectra of imidazoles 3a–h are characterized by prominent absorption maxima with *λ*_max_ between 322 and 350 nm, while oxazoles 5a–c have *λ*_max_ between 327 and 331 nm. Upon exposure to trifluoromethanesulfonic acid (TfOH), bathochromic shifts of 11–43 nm are observed (Fig. 6, blue and red curve) for the more basic imidazoles (p*K*_a_(BH^+^) ∼7.0); these shifts are fully reversible. In contrast, the less basic oxazoles (p*K*_a_(BH^+^) ∼0.8) are not protonated and their exposure to TfOH results in negligible changes in their UV/vis absorption (Fig. 6, green and magenta curve). Fluorescence images (insert in Fig. 6) similarly show the response of 3a to protonation and miniscule response of 5a. Computational and spectroscopic analyses (Fig. S52 and S53) suggest that a fourfold protonation occurs on tetraimidazoles, indicating the independence of protonation sites from one another.

## Conclusions

In conclusion, we have developed a general and facile synthetic protocol to access inverted oxazolophane and imidazolophane macrocycles. The divergent positioning of free azole valences in these molecules, as well as their shape-persistent nature and limited conformational flexibility, suggest that these species may become privileged 90° motifs in the construction of ordered materials, such as *e.g.*, metal–organic or covalent organic frameworks,^23^ or molecular cages. While their aromaticity is dominated by the local five- and six-membered ring currents, multielectron oxidation or reduction of inverted azolophanes could result in globally (anti)aromatic systems. We are exploring several of these directions and will report our findings in due course.

## Author contributions

Y.-H. L. synthesized and characterized all new compounds, and obtained diffraction quality crystals of 3b–d, 3h, and 5a. X. W. solved crystal structures of 3b–d, 3h, and 5a. D. W. S. and P. A. S. performed the computational analysis and secured part of the funding. O. Š. M. obtained funding for the US side of this collaboration and wrote the paper incorporating the input from all authors.

## Conflicts of interest

There are no conflicts to declare.

## Supplementary Material

SC-OLF-D5SC05981J-s001

SC-OLF-D5SC05981J-s002

## Data Availability

The experimental data supporting this article (synthetic procedures and spectroscopic and crystallographic characterization data) have been included as part of the Supplementary Information (SI). CCDC 2478993–2478997 (3b, 3c, 3d, 3h, and 5a) contain the supplementary crystallographic data for this paper.^24*a*–*e*^
