# Supplementary material for: Inverted azolophanes: alternant o-heteroarene/p-arene macrocycles
Source: Chem Sci. 2025 Sep 23;16(42):19967–72. doi: 10.1039/d5sc05981j (PMC12477710; doi:10.1039/d5sc05981j)
Supplement: SC-016-D5SC05981J-s001 [file SC-016-D5SC05981J-s001.pdf]

Supplementary Information (SI) for Chemical Science.
This journal is © The Royal Society of Chemistry 2025

Inverted Azolophanes: Alternant *o*-Heteroarene/*p*-Arene Macrocycles

Yun-Hsien Lin,[a] Xiqu Wang,[a] Dariusz W. Szczepanik,*[b] Paweł A. Wieczorkiewicz,[c] and Ognjen Š. Miljanić*[a],[d]

[a] Department of Chemistry, University of Houston, 112 Fleming Building, Houston, Texas 77204-5003, United States

[b] K. Gumiński Department of Theoretical Chemistry, Faculty of Chemistry, Jagiellonian University, Gronostajowa 2, 30-387 Kraków, Poland

[c] Faculty of Chemistry, Warsaw University of Technology, Noakowskiego 3, 00-664 Warszawa, Poland

[d] Faculty of Chemical Engineering, Industrial University of Ho Chi Minh City, Ho Chi Minh City 71408, Vietnam

Email: dariusz.szczepanik@uj.edu.pl, miljanic@uh.edu

Supporting Information

General Methods and Materials S2

Syntheses of New Compounds S3

^{1}H and ^{13}C NMR Spectra of New Compounds S10

Single Crystal Structures and Analyses S26

UV-Vis Spectroscopic Titrations S34

Computational Analysis of Protonation S41

References S43

General Methods and Materials

All reactions were performed under a nitrogen atmosphere. Reagents bought from commercial suppliers were used without further purification. Reagents were purchased from the following suppliers: terephthalaldehyde (TCI Chemical), sodium cyanide (Oakwood), 2-methoxyethanol (Oakwood), dimethyl sulfoxide (Fisher Scientific), acetic anhydride (Sigma Aldrich), sulfuric acid (Oakwood), nitric acid (VWR), acetic acid (VWR), ammonium acetate (EMD), 3,4-dihydroxybenzaldehyde (Oakwood), 3-hydroxybenzaldehyde (Sigma Aldrich), 4-hydroxybenzaldehyde (Sigma Aldrich), benzaldehyde (Alfa Aesar), 4-fluorobenzaldehyde (Sigma Aldrich), 4-(hydroxymethyl)benzaldehyde (AOBChem), 4-anisaldehyde (TCI), and 9-anthraldehyde (Sigma Aldrich). Cyclotetrabenzoin,[1] cyclotetrabenzil (**1**),[2] and esterified cyclotetrabenzoins **4a–c**[3] were synthesized using literature procedures.

^{1}H spectra were collected on JEOL ECA-400 MHz, JEOL ECA-500 MHz, and JEOL ECA-600 MHz spectrometers with working frequencies of 400 MHz, 500 MHz, and 600 MHz, respectively, for ^{1}H nuclei. Chemical shifts are given in ppm (δ) with respect to the solvent peak or tetramethylsilane as a standard for ^{1}H NMR spectra. NMR spectra were collected at 25 °C, unless otherwise indicated. Infrared spectra were collected on a Nicolet iS10 FT-IR spectrometer. Mass spectrometry analyses were measured by the Mass Spectrometry Facility at the University of Texas at Austin. Melting points were measured in open capillary tubes using a Barnstead International Mel-TEMP and a DigitMelt apparatus and are uncorrected. UV-Vis spectra were measured on a PerkinElmer LAMBDA 25 UV/Vis spectrophotometer.

Experiments are presented in the order following the discussion of the manuscript.

Compound numbers are identical to those in the main text of the manuscript.

Syntheses of New Compounds

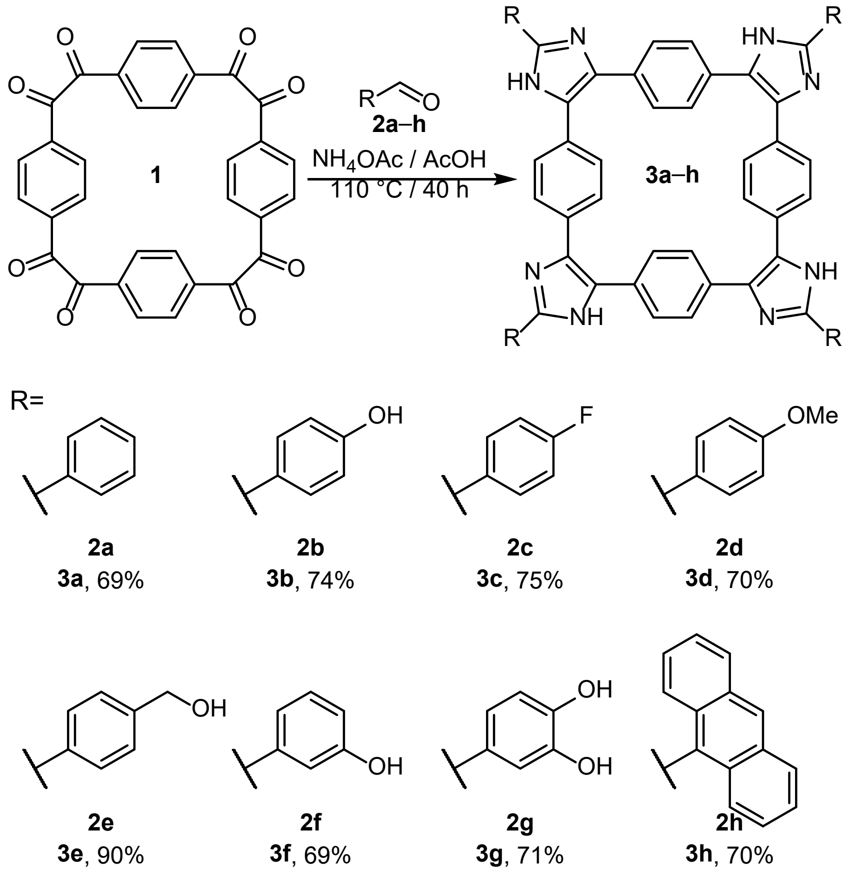

Figure S1. Synthetic condition and the substrates for the preparation of imidazole macrocycles **3a**–**h**.

<u>General Procedure for Imidazole-Based Macrocycles **3a**–**h**</u>

A suspension of compound **1**[3] (231 mg, 0.44 mmol), the appropriate arylaldehyde (**2a**–**h**, 1.84 mmol, 4.2 equiv.), and NH_4OAc (1.01 g, 0.013 mol) in AcOH (3.0 mL) was heated at 110 °C for 40 h. The reaction mixture was cooled to 20 °C and deionized H_2O (10 mL) was added. The resulting mixture was filtered to obtain the crude product, which was suspended in PhMe (50 mL) and then heated at 80 °C for 14 h. The resulting mixture was filtered, and the solid was rinsed with PhMe (20 mL) and Et_2O (20 mL) to afford tetraimidazoles **3a**–**h** in yields ranging between 69 and 90%.

Compound 2,9,16,23-tetraphenyl-1,8,15,22-tetrahydro-4,7:11,14:18,21:25,28-tetraethenocyclo-tetracosa[1,2-d:7,8-d':13,14-d'':19,20-d''']tetraimidazole (**3a**)

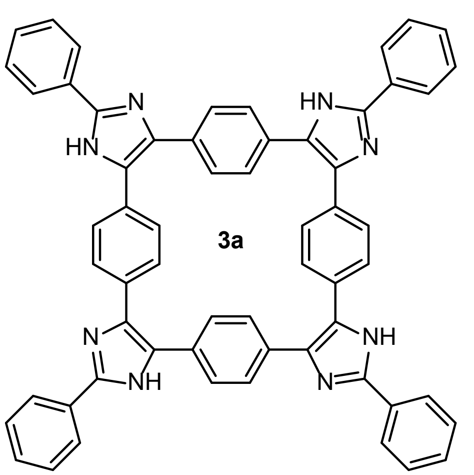

Yield: 69%; yellow solid; m.p. >250 °C. ^{1}H NMR (400 MHz, DMSO-d_6): δ 8.08 (d, J = 7.6 Hz, 8H, ArH), 7.69 (s, 16H, ArH), 7.48 (t, J = 7.2 Hz, 8H, ArH), 7.37 ppm (t, J = 6.5 Hz, 4H, ArH). ^{13}C NMR (126 MHz, DMSO-d_6): δ 137.02, 135.50, 134.16, 130.77, 130.63, 130.00, 129.24, 125.97 ppm. FT-IR (neat): 3060, 1665, 1607, 1571, 1499, 1429, 1405, 1296, 1201, 1127, 968, 832, 774, 715, 687, 523 cm^{-1}. ESI-HRMS: m/z [M+H]$^+$: Calcd for [$C_{60}H_{40}N_8 \cdot H$]$^+$: 873.3466; Found: 873.3449.

Compound 2,9,16,23-tetra(4-hydroxyphenyl)-1,8,15,22-tetrahydro-4,7:11,14:18,21:25,28-tetraetheno-cyclo-tetracosa[1,2-d:7,8-d':13,14-d'':19,20-d''']tetraimidazole (**3b**)

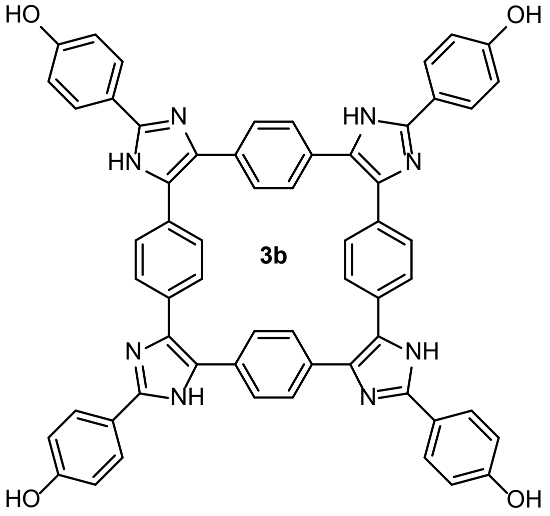

Yield: 74%; yellow solid; m.p. >450 °C. ^{1}H NMR (400 MHz, DMSO-d_6)**:** δ 9.85 (s, 4H, OH), 7.89 (d, J = 8.5 Hz, 8H, ArH), 7.65 (s, 16H, ArH), 6.85 ppm (d, J = 8.7 Hz, 8H, ArH). ^{13}C NMR (126 MHz, DMSO-d_6): δ 158.73, 146.88, 136.17, 131.29, 130.03, 127.92, 127.87, 115.99 ppm. FT-IR (neat): 3058, 1666, 1607, 1498, 1397, 1198, 1114, 965, 834, 720, 606, 524 cm^{-1}. ESI-HRMS: m/z [M+H]$^+$: Calcd for [$C_{60}H_{40}N_8O_4 \cdot H$]$^+$: 937.3264; Found: 937.3245. Diffraction-quality single crystals of **3b** were grown by vapor diffusion of *n*-pentane into the solution of **3b** in 1,4-dioxane and *N,N*-diethylformamide (DEF) over 10 days.

Compound 2,9,16,23-tetra(4-fluorophenyl)-1,8,15,22-tetrahydro-4,7:11,14:18,21:25,28-tetraetheno-cyclo-tetracosa[1,2-d:7,8-d':13,14-d'':19,20-d''']tetraimidazole (**3c**)

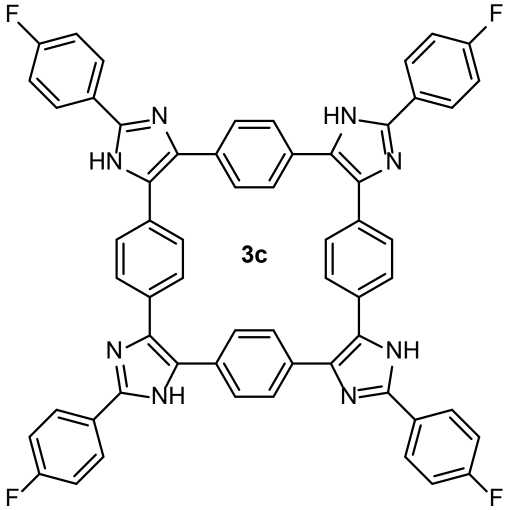

Yield: 75%; yellow solid; m.p. >250 °C. ^{1}H NMR (400 MHz, DMSO-d_6): δ 8.12–8.09 (m, 8H, ArH), 7.67 (s, 16H, ArH), 7.31 ppm (t, J = 7.7 Hz, 8H, ArH). ^{13}C NMR (126 MHz, DMSO-d_6): δ 163.68, 161.74, 145.65, 130.01, 128.06, 127.43, 116.28, 116.10 ppm. ^{19}F NMR (376 MHz, DMSO-d_6): δ −113.2 ppm. FT-IR (neat): 3269, 1666, 1601, 1497, 1299, 1215, 964, 833, 721, 614, 513 cm^{-1}. ESI-HRMS: *m/z* [M+H]$^+$: Calcd for [$C_{60}H_{36}F_4N_8$·H]$^+$: 945.3094; Found: 945.3072. Diffraction-quality single crystals of **3c** were grown via vapor diffusing of MeOH into the solution of **3c** in DEF over 5 days.

Compound 2,9,16,23-tetra(4-methoxyphenyl)-1,8,15,22-tetrahydro-4,7:11,14:18,21:25,28-tetraetheno-cyclo-tetracosa[1,2-d:7,8-d':13,14-d'':19,20-d''']tetraimidazole (**3d**)

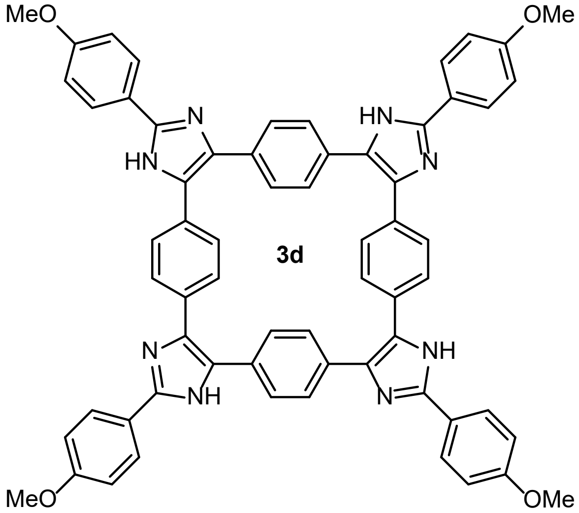

Yield: 70%; green solid; m.p. >250 °C. ^{1}H NMR (400 MHz, DMSO-d_6): δ 8.01 (d, J = 8.7 Hz, 8H, ArH), 7.67 (s, 16H, ArH), 7.04 (d, J = 8.5 Hz, 8H, ArH), 3.79 ppm (s, 12H, CH_3). ^{13}C NMR (126 MHz, DMSO-d_6): δ 146.45, 142.23, 130.63, 130.56, 127.46, 123.53, 123.50, 114.62, 55.77 ppm. FT-IR (neat): 3059, 1665, 1612, 1571, 1502, 1429, 1404, 1292, 1250, 1202, 1182, 1076, 1030, 972, 931, 886, 863, 830, 715, 522 cm^{-1}. ESI-HRMS: *m/z* [M+H]$^+$: Calcd for [$C_{64}H_{48}N_8O_4$·H]$^+$: 993.3874; Found: 993.3871. Diffraction-quality single crystals of **3d** were grown by vapor diffusion of MeOH into the solution of **3d** in 2-methoxyethanol over 7 days.

Compound 2,9,16,23-tetra(4-acetyloxymethylphenyl)-1,8,15,22-tetrahydro-4,7:11,14:18,21:25,28-tetraethenocyclo-tetracosa[1,2-d:7,8-d':13,14-d'':19,20-d''']tetraimidazole (**3e**)

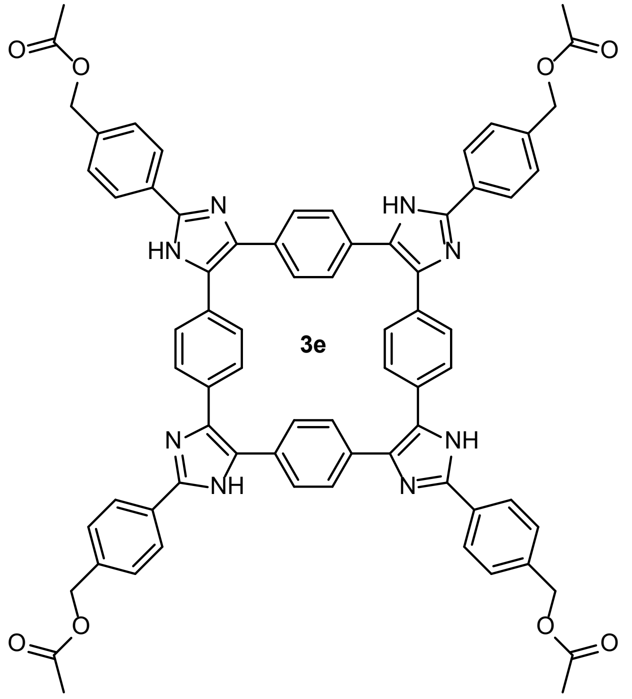

Yield: 90%; yellow solid; m.p. >250 °C. ^{1}H NMR (400 MHz, DMSO-d_6): δ 8.08 (d, J = 8.3 Hz, 8H, ArH), 7.68 (s, 16H, ArH), 7.46 (d, J = 8.3 Hz, 8H, ArH), 5.10 (s, 8H, CH_2), 2.07 ppm (s, 12H, CH_3). ^{13}C NMR (126 MHz, DMSO-d_6): δ 170.86, 146.09, 136.77, 130.40, 130.39, 128.88, 127.93, 126.01, 126.00, 65.76, 21.31 ppm. FT-IR (neat): 3325, 1713, 1502, 1372, 1253, 1113, 1028, 961, 832, 670, 603, 514 cm^{-1}. ESI-HRMS: *m/z* [M+H]$^+$: Calcd for [$C_{72}H_{56}N_8O_8 \cdot H$]$^+$: 1161.4275; Found: 1161.4294.

Compound 2,9,16,23-tetra(3-hydroxyphenyl)-1,8,15,22-tetrahydro-4,7:11,14:18,21:25,28-tetraetheno-cyclo-tetracosa[1,2-d:7,8-d':13,14-d'':19,20-d''']tetraimidazole (**3f**)

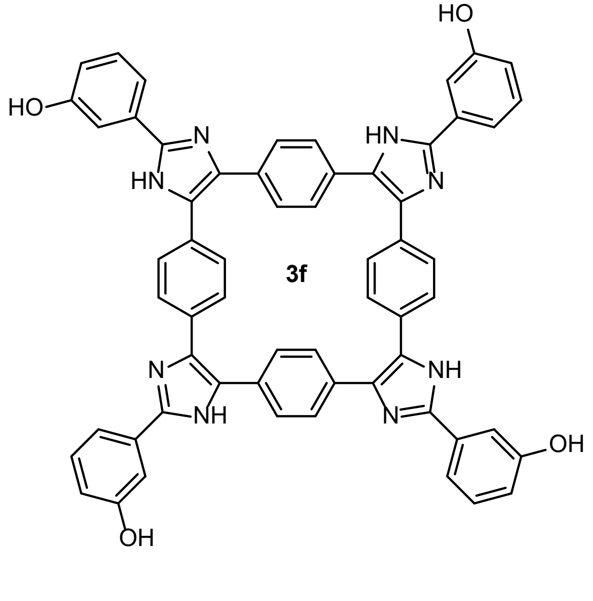

Yield: 69%; yellow solid; m.p. >450 °C. ^{1}H NMR (500 MHz, DMSO-d_6): δ 9.61 (s, 4H, OH), 7.86 (s, 4H, ArH), 7.70 (s, 16H, ArH), 7.52 (d, J = 8.2 Hz, 8H, ArH), 7.27 (t, J = 7.1 Hz, 4H, ArH), 6.78 ppm (d, J = 6.2 Hz, 4H, ArH). ^{13}C NMR (126 MHz, DMSO-d_6): δ 158.12, 136.35, 132.03, 130.96, 130.26, 122.36, 121.66, 116.85, 116.08, 112.87 ppm. FT-IR (neat): 3062, 1664, 1593, 1475, 1409, 1304, 1202, 1112, 970, 844, 786, 712, 531, 459 cm^{-1}. ESI-HRMS: *m/z* [M+H]$^+$: Calcd for [$C_{60}H_{40}N_8O_4 \cdot H$]$^+$: 937.3275; Found: 937.3245.

Compound 2,9,16,23-tetra(3,4-dihydroxyphenyl)-1,8,15,22-tetrahydro-4,7:11,14:18,21:25,28-tetra-ethenocyclo-tetracosa[1,2-d:7,8-d':13,14-d'':19,20-d''']tetraimidazole (**3g**)

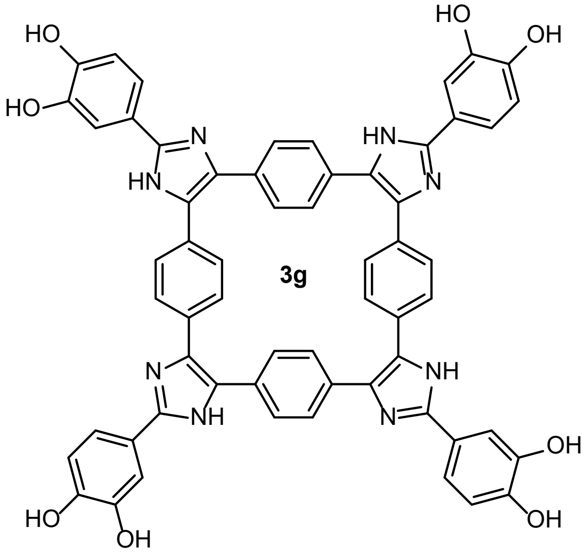

Yield: 71%; black solid; m.p. >250 °C. ^{1}H NMR (400 MHz, DMSO-d_6): δ 9.22 (s, 4H, OH), 9.11 (s, 4H, OH), 7.66 (s, 16H, ArH), 7.51 (s, 4H, ArH), 7.36 (d, *J* = 9.2 Hz, 4H, ArH), 6.80 ppm (d, *J* = 8.3 Hz, 8H, ArH). ^{13}C NMR (126 MHz, DMSO-d_6): δ 147.14, 146.82, 145.90, 130.02, 127.87, 122.26, 122.15, 117.82, 116.15, 113.90 ppm. FT-IR (neat): 3160, 1655, 1604, 1509, 1401, 1273, 1192, 705, 521 cm^{-1}. ESI-HRMS: *m/z* $[M+2H]^{2+}/2$: Calcd for $[C_{60}H_{40}N_8O_8{\cdot}2H]^{2+}/2$: 501.1576; Found: 501.1557.

Compound 2,9,16,23-tetra(9-anthracenyl)-1,8,15,22-tetrahydro-4,7:11,14:18,21:25,28-tetraetheno-cyclo-tetracosa[1,2-d:7,8-d':13,14-d'':19,20-d''']tetraimidazole (**3h**)

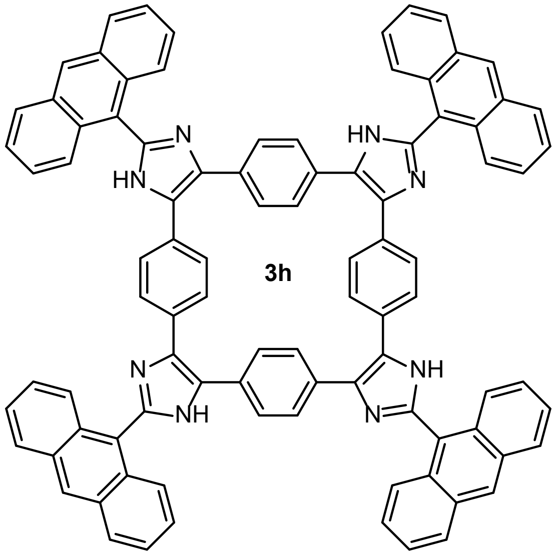

Yield: 70%; brown solid; m.p. >250 °C. ^{1}H NMR (500 MHz, DMSO-d_6): δ 8.77 (s, 4H, ArH), 8.17–8.15 (m, 8H, ArH), 7.97–7.89 (m, 16H, ArH), 7.87–7.77 (m, 8H, ArH), 7.55 ppm (t, *J* = 3.9 Hz, 16H, ArH). ^{13}C NMR (126 MHz, DMSO-d_6): δ 164.76, 154.35, 144.30, 135.11, 131.41, 131.30, 128.96, 128.84, 127.89, 127.24, 126.50, 126.13 ppm. FT-IR (neat): 3050, 1666, 1531, 1416, 1327, 1200, 841, 729, 644, 590, 525 cm^{-1}. ESI-HRMS: *m/z* $[M+H]^{+}$: Calcd for $[C_{92}H_{56}N_8{\cdot}H]^{+}$: 1273.4701; Found: 1273.4736. Diffraction-quality single crystals of **3h** were grown by vapor diffusion of *n*-pentane into the solution of **3h** in 1,4-dioxane over 3 days.

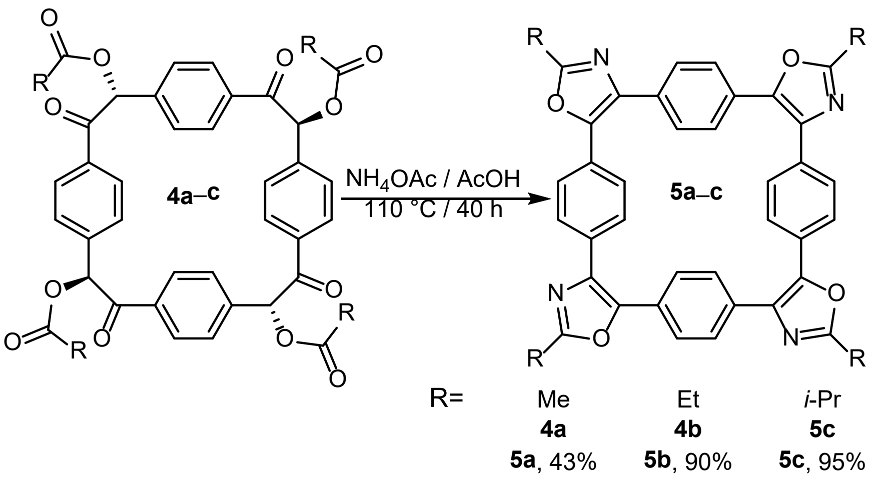

Figure S2. Synthetic conditions and the substrates for the preparation of oxazole macrocycles **5a**–**c**.

Compound **4a**–**c**[2] (1.00 g, 1.22–1.44 mmol) and NH_4OAc (1.80 g, 23.4 mmol, 16.2 equiv.) were suspended in AcOH (10 mL) and heated at 110 °C for 40 h. The reaction mixture was cooled to 20 °C and filtered. The obtained solid was rinsed with deionized H_2O (20 mL), EtOH (20 mL), and Et_2O (20 mL) to obtain the crude product, which was recrystallized from PhMe to afford compounds **5a**–**c** as yellow solids in yields ranging from 43 to 95%.

<u>Compound 2,9,16,23-tetramethyl-4,7:11,14:18,21:25,28-tetraethenocyclotetracosa[1,2-d:7,8-d':13,14-d'':19,20-d''']tetrakis(oxazole)) (**5a**)</u>

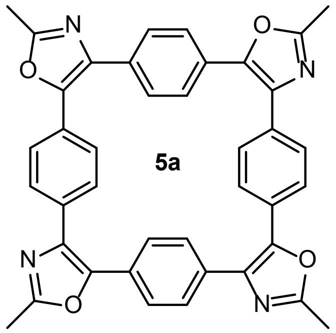

Yield: 43%; yellow solid; m.p. >450 °C. ^{1}H NMR (400 MHz, $CDCl_3$): δ 7.80 (d, J=8.0 Hz, 8H, ArH), 7.73–7.70 (m, 8H, ArH), 2.56 ppm (s, 12H, CH_3). ^{13}C NMR (151 MHz, $CDCl_3$): δ 160.78, 145.73, 134.81, 132.72, 129.12, 128.05, 126.59, 14.12 ppm. FT-IR (neat): 3038, 1710, 1588, 1498, 1418, 1276, 1217, 1052, 960, 837, 741, 671, 580, 524, 441 cm^{-1}. ESI-HRMS: m/z $[M+H]^+$: Calcd for $[C_{40}H_{28}N_4O_4{\cdot}H]^+$: 629.2183; Found: 629.2211. Diffraction-quality single crystals of **5a** were grown by vapor diffusion of Et_2O into the solution of **5a** in 1,2-dichloroethane over 5 days.

Compound 2,9,16,23-tetraethyl-4,7:11,14:18,21:25,28-tetraethenocyclotetracosa[1,2-d:7,8-d':13,14-d'':19,20-d''']tetrakis(oxazole)) (**5b**)

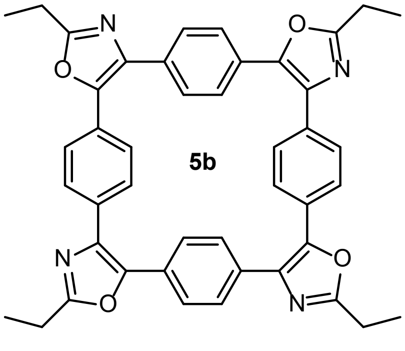

Yield: 90%; yellow solid; m.p. >260 °C. ^{1}H NMR (400 MHz, $CDCl_3$): δ 7.81 (d, J = 8.3 Hz, 8H, ArH), 7.73-7.70 (m, 8H, ArH), 2.88 (q, J = 7.5 Hz, 8H, CH_2), 1.42 (t, J = 7.6 Hz, 12H, CH_3) ppm. ^{13}C NMR (126 MHz, $CDCl_3$): δ 165.12, 145.50, 134.68, 132.80, 128.45, 128.12, 126.58, 21.84, 11.48ppm. FT-IR (neat): 3203, 2979, 1673, 1607, 1580, 1503, 1412, 1214, 1057, 966, 842, 589 cm^{-1}. Diffraction-quality single crystals of **5b** were grown by vapor diffusion of MeOH into the solution of **5b** in 1,2-dichloroethane over 4 days.

Compound 2,9,16,23-tetra(1-methylethyl)-4,7:11,14:18,21:25,28-tetraethenocyclotetracosa[1,2-d:7,8-d':13,14-d'':19,20-d''']tetrakis(oxazole)) (**5c**)

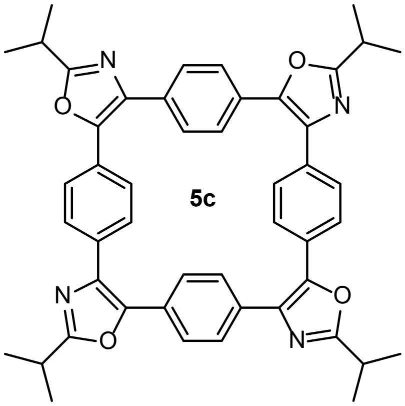

Yield: 95%; yellow solid; m.p. >260 °C. ^{1}H NMR (600 MHz, $CDCl_3$) δ 7.81 (d, J = 7.6 Hz, 8H), 7.72 (t, J = 8.6 Hz, 8H), 3.17 (sept, J = 6.9 Hz, 4H), 1.43 (d, J = 6.9 Hz, 24H) ppm. ^{13}C NMR (126 MHz, $CDCl_3$): δ 168.21, 145.26, 134.58, 132.88, 128.48, 128.17, 126.56, 28.63, 20.64 ppm. FT-IR (neat): 2973, 2936, 1701, 1607, 1575, 1501, 1410, 1218, 1057, 965, 842, 743, 595, 543 cm^{-1}.

^{1}H and ^{13}C NMR Spectra of New Compounds

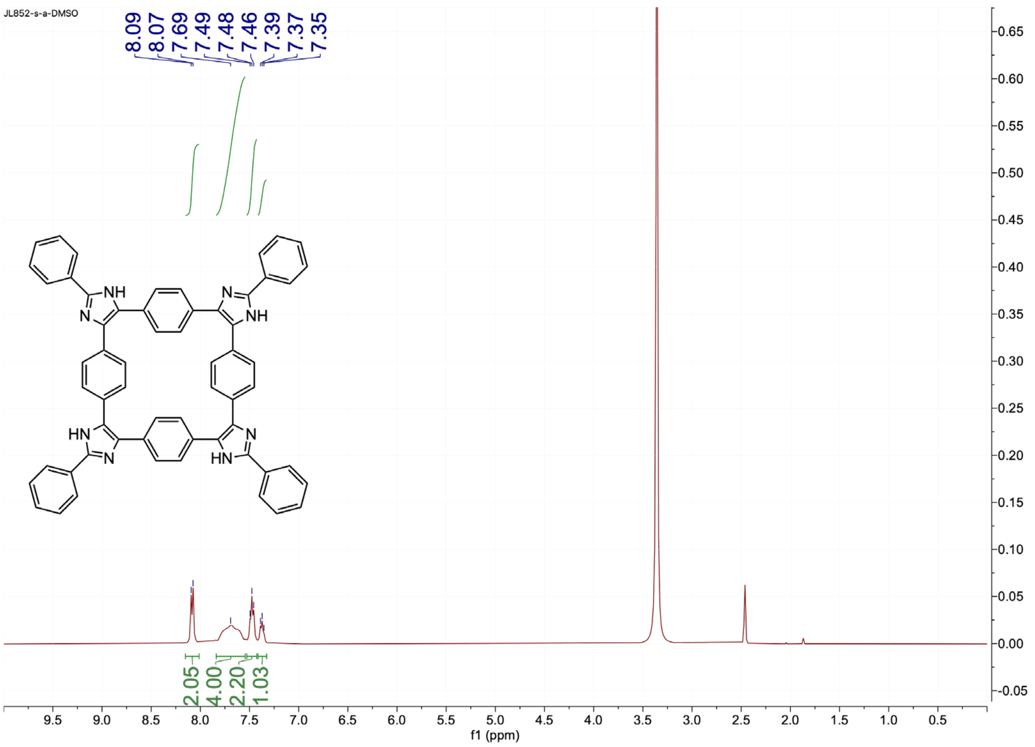

Figure S3. ^{1}H NMR spectrum of compound **3a** (400 MHz, DMSO-d_6, 25 °C).

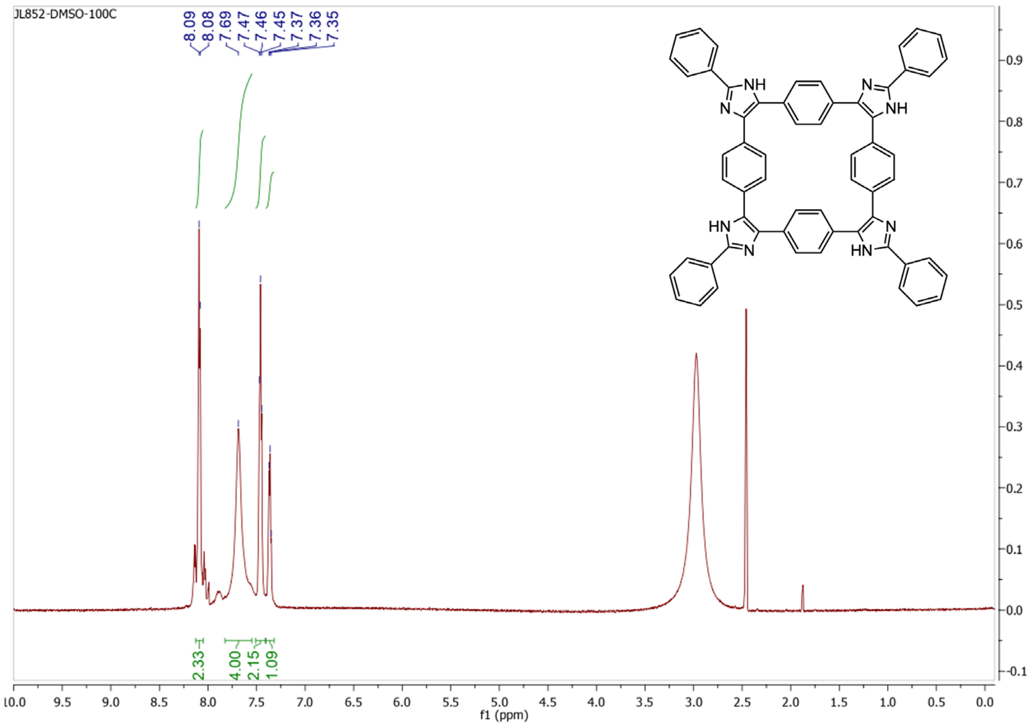

Figure S4. ^{1}H NMR spectrum of compound **3a** (600 MHz, DMSO-d_6, 100 °C).

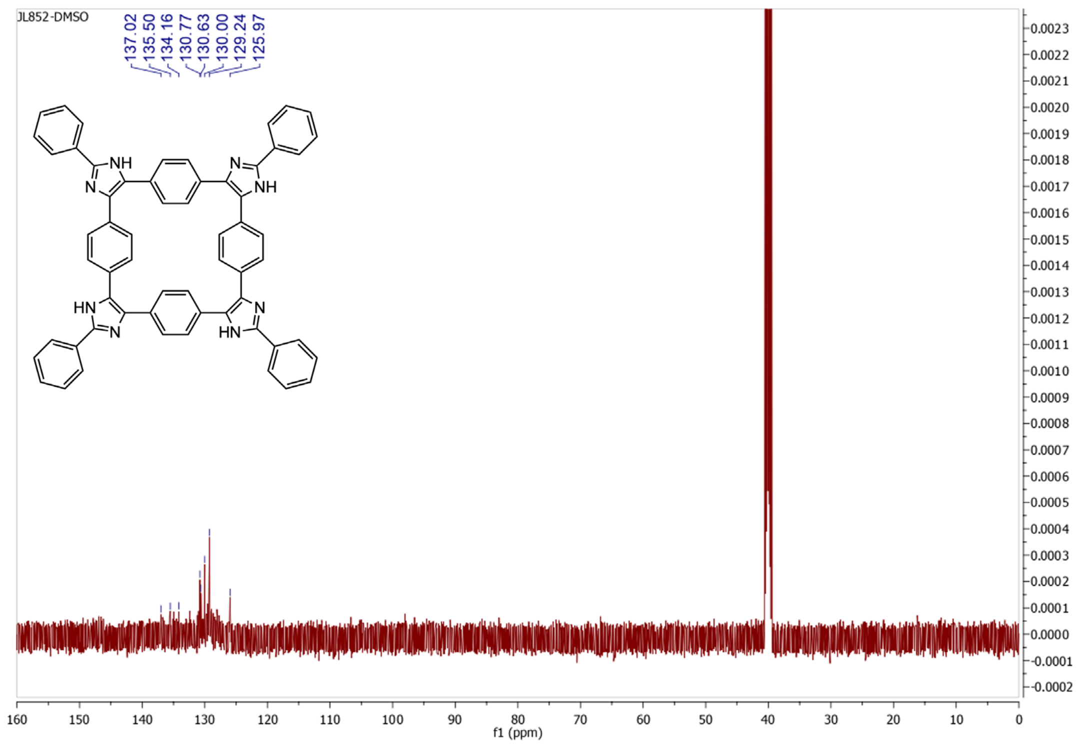

Figure S5. ^{13}C NMR spectrum of compound **3a** (126 MHz, DMSO-d_6, 25 °C).

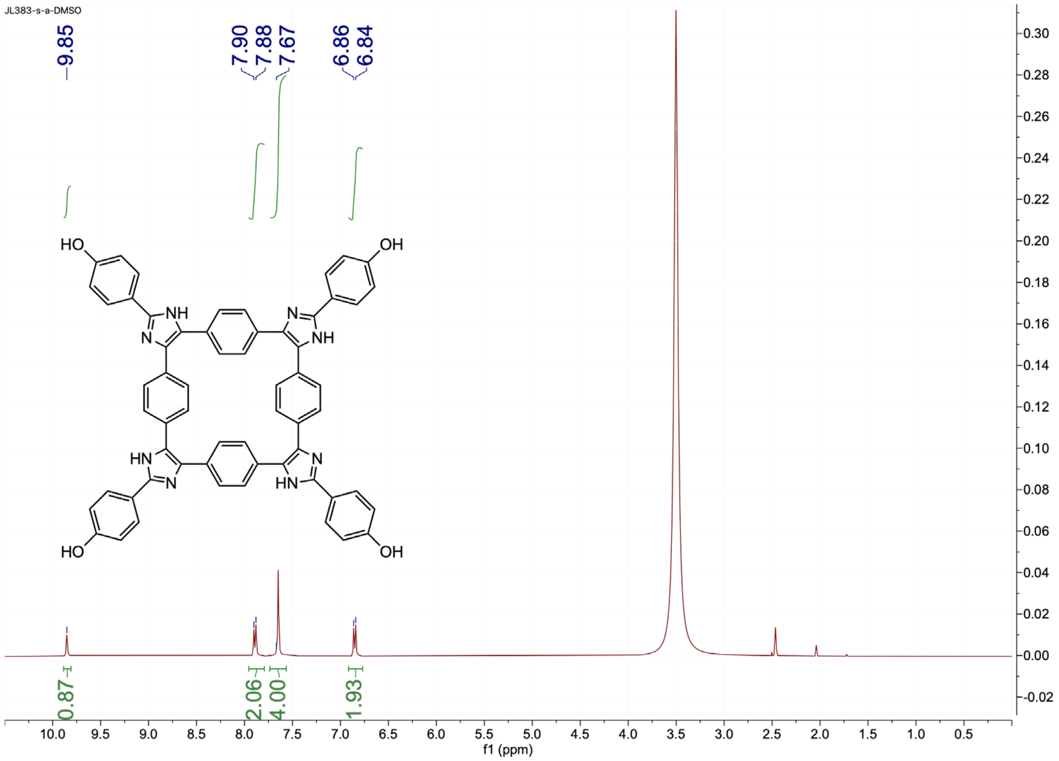

Figure S6. ^{1}H NMR spectrum of compound **3b** (400 MHz, DMSO-d_6, 25 °C).

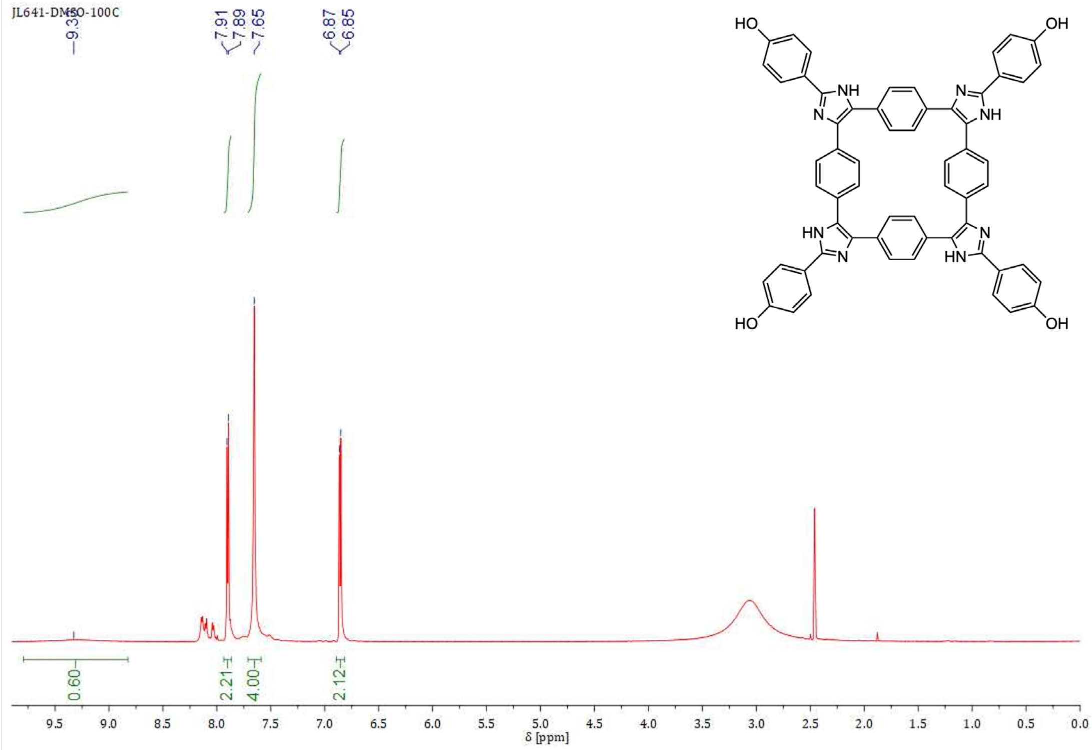

Figure S7. ^{1}H NMR spectrum of compound **3b** (600 MHz, DMSO-d_6, 100 °C).

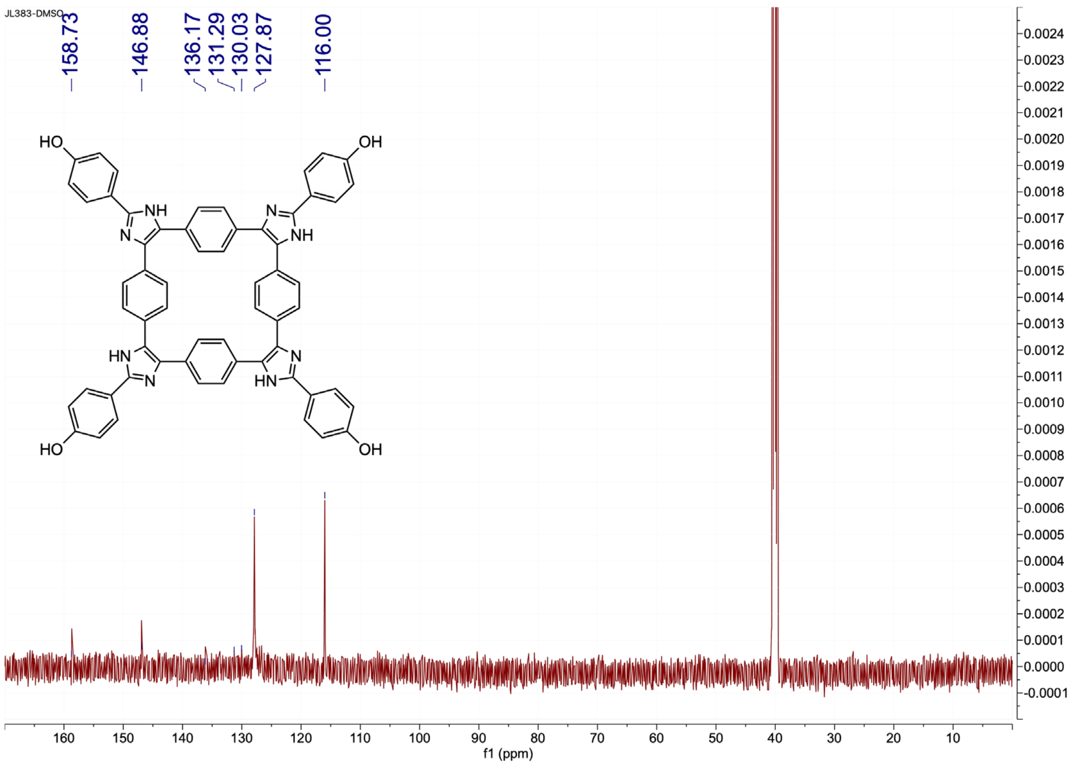

Figure S8. ^{13}C NMR spectrum of compound **3b** (126 MHz, DMSO-d_6, 25 °C).

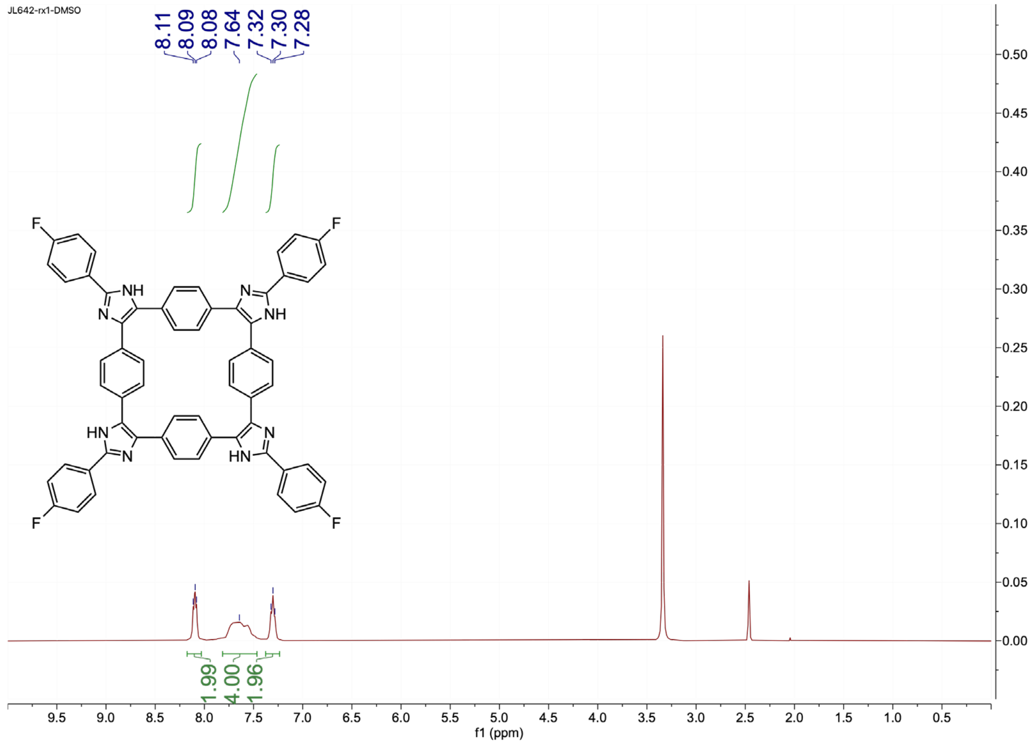

Figure S9. ^{1}H NMR spectrum of compound **3c** (400 MHz, DMSO-d_6, 25 °C).

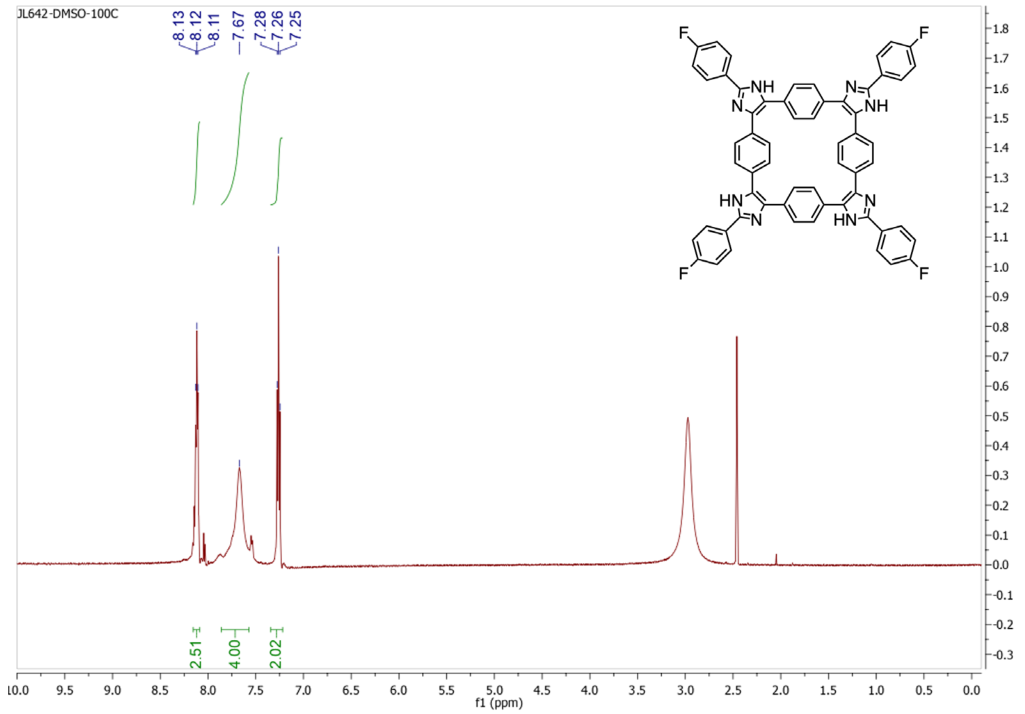

Figure S10. ^{1}H NMR spectrum of compound **3c** (600 MHz, DMSO-d_6, 100 °C).

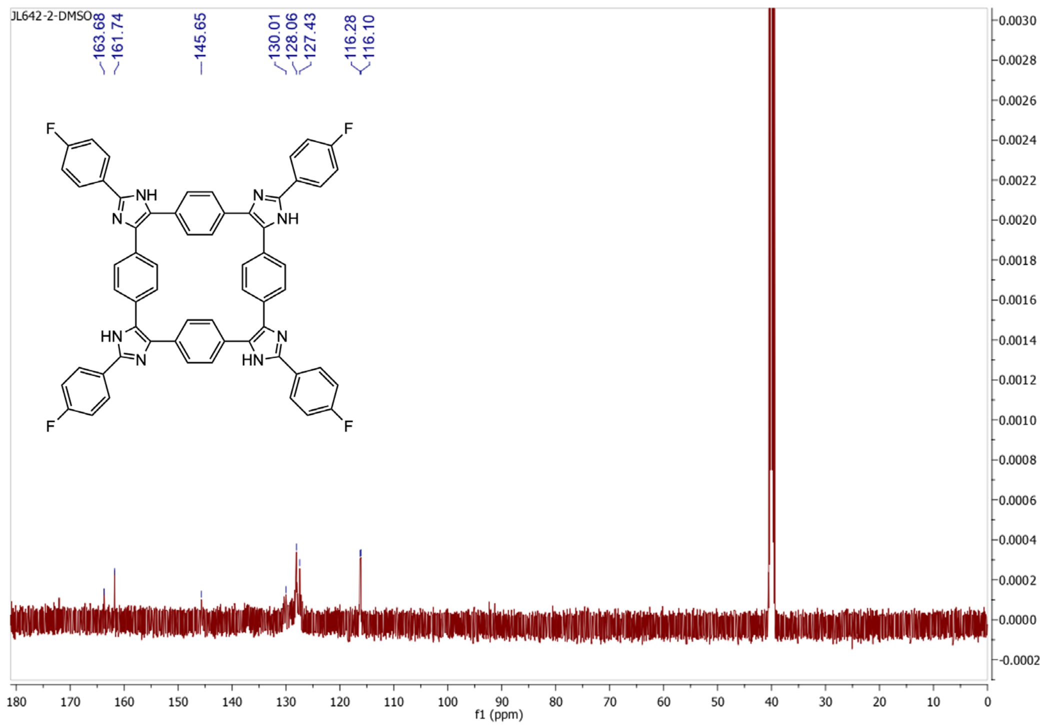

Figure S11. ^{13}C NMR spectrum of compound **3c** (126 MHz, DMSO-d_6, 25 °C).

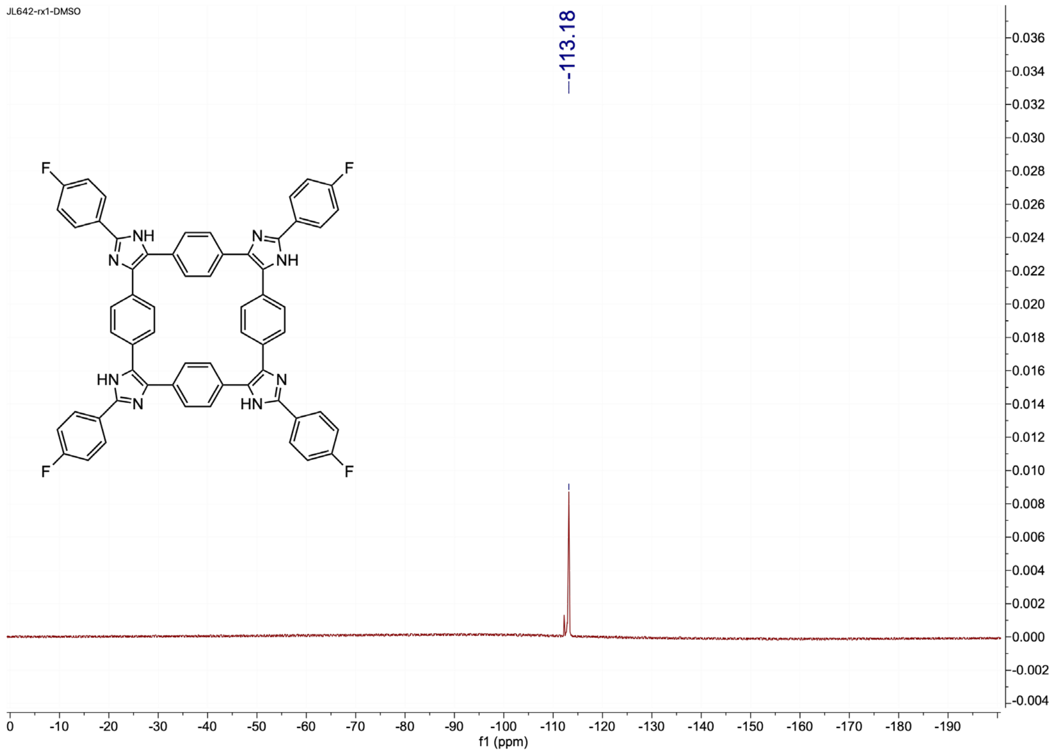

Figure S12. ^{19}F NMR spectrum of compound **3c** (376 MHz, DMSO-d_6, 25 °C).

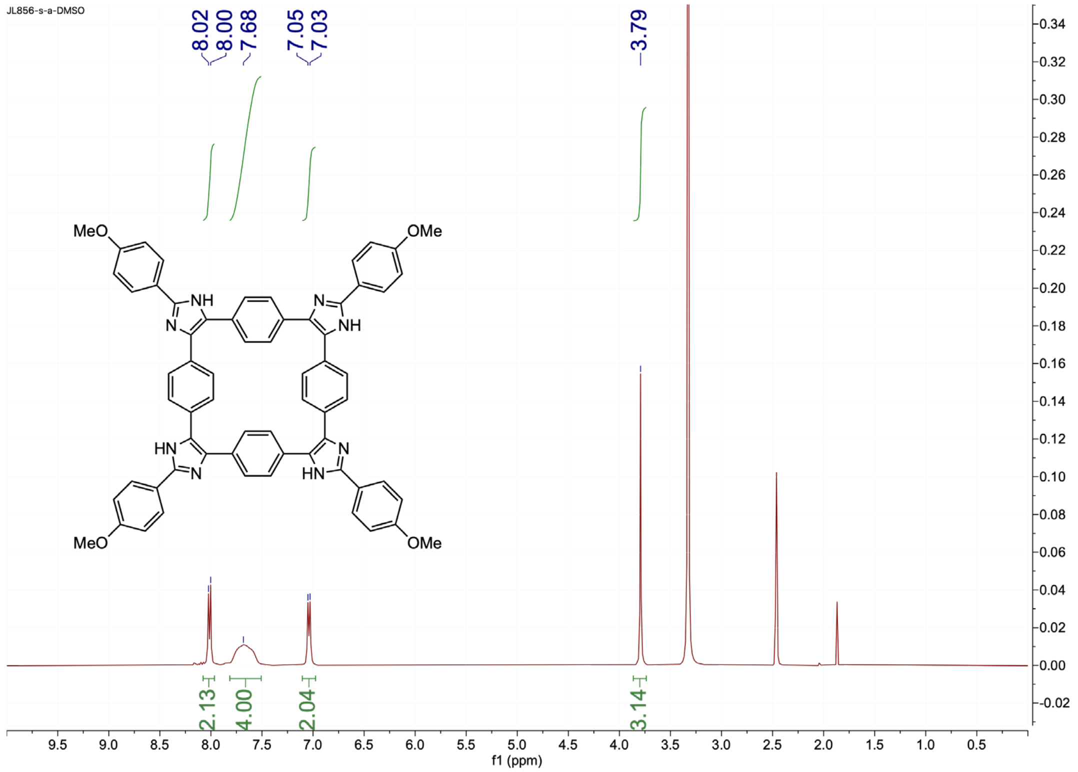

Figure S13. ^{1}H NMR spectrum of compound **3d** (400 MHz, DMSO-d_6, 25 °C).

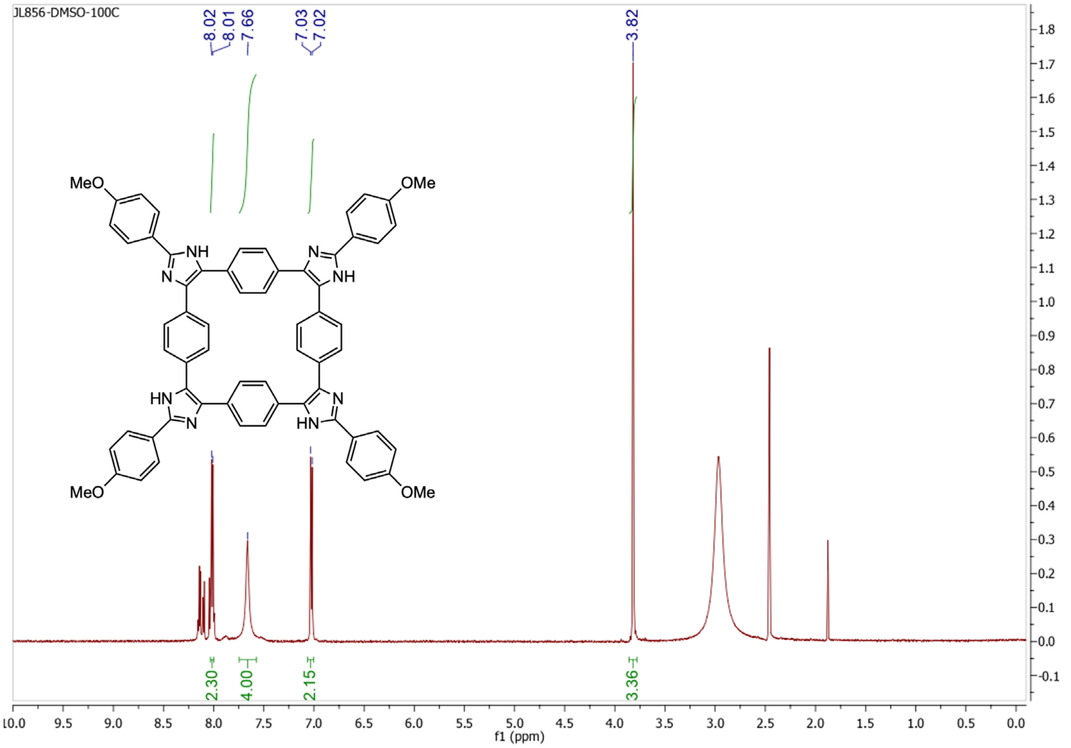

Figure S14. ^{1}H NMR spectrum of compound **3d** (600 MHz, DMSO-d_6, 100 °C).

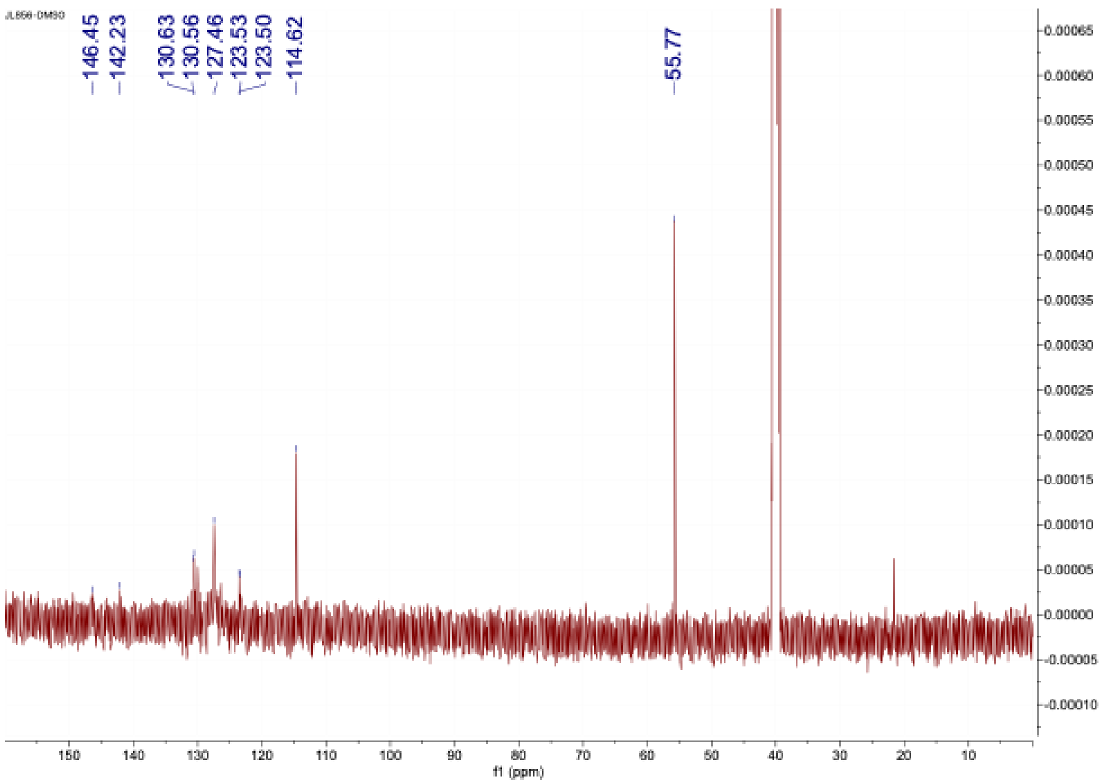

Figure S15. ^{13}C NMR spectrum of compound **3d** (126 MHz, DMSO-d_6, 25 °C).

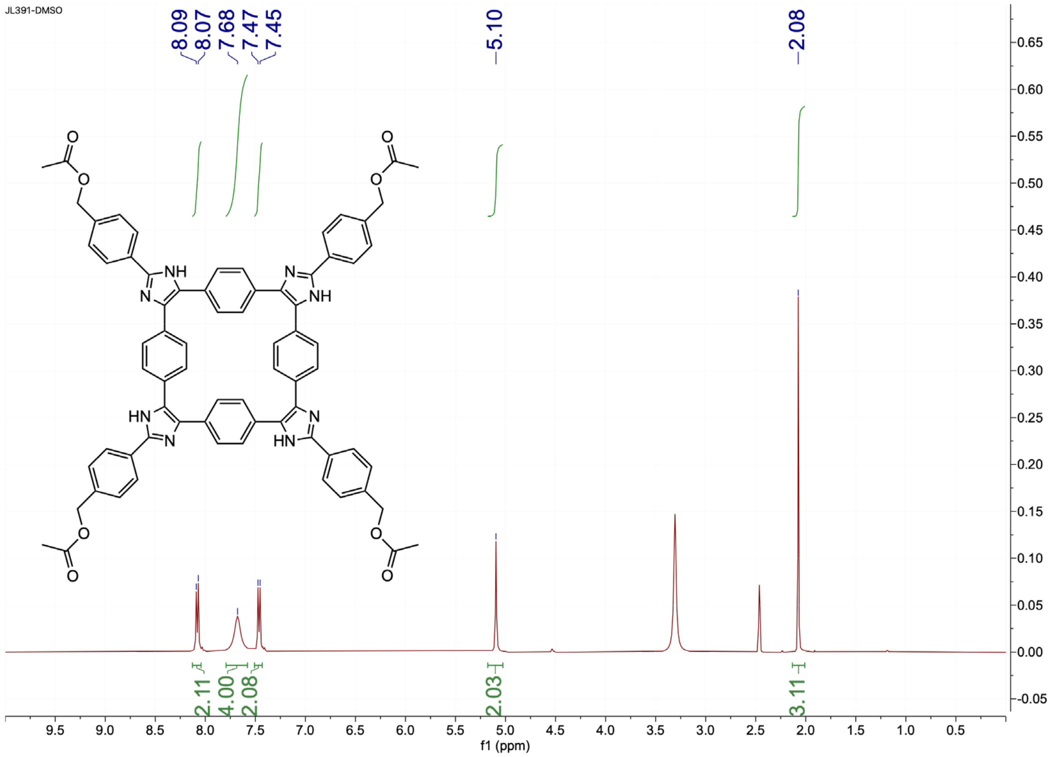

Figure S16. ^{1}H NMR spectrum of compound **3e** (400 MHz, DMSO-d_6, 25 °C).

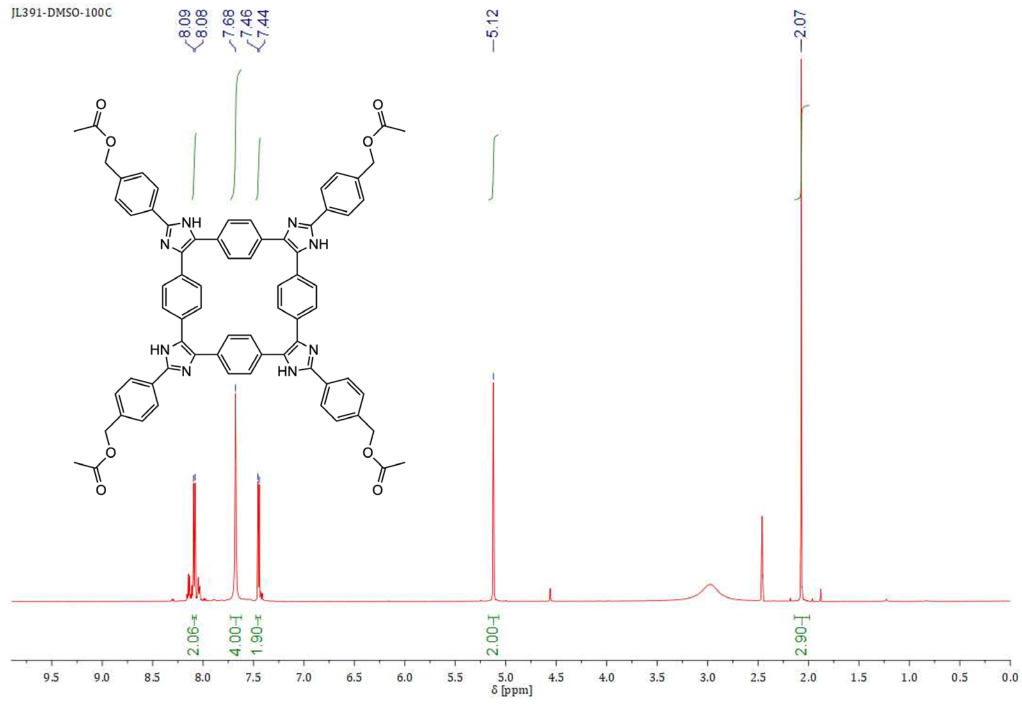

Figure S17. ^{1}H NMR spectrum of compound **3e** (600 MHz, DMSO-d_6, 100 °C).

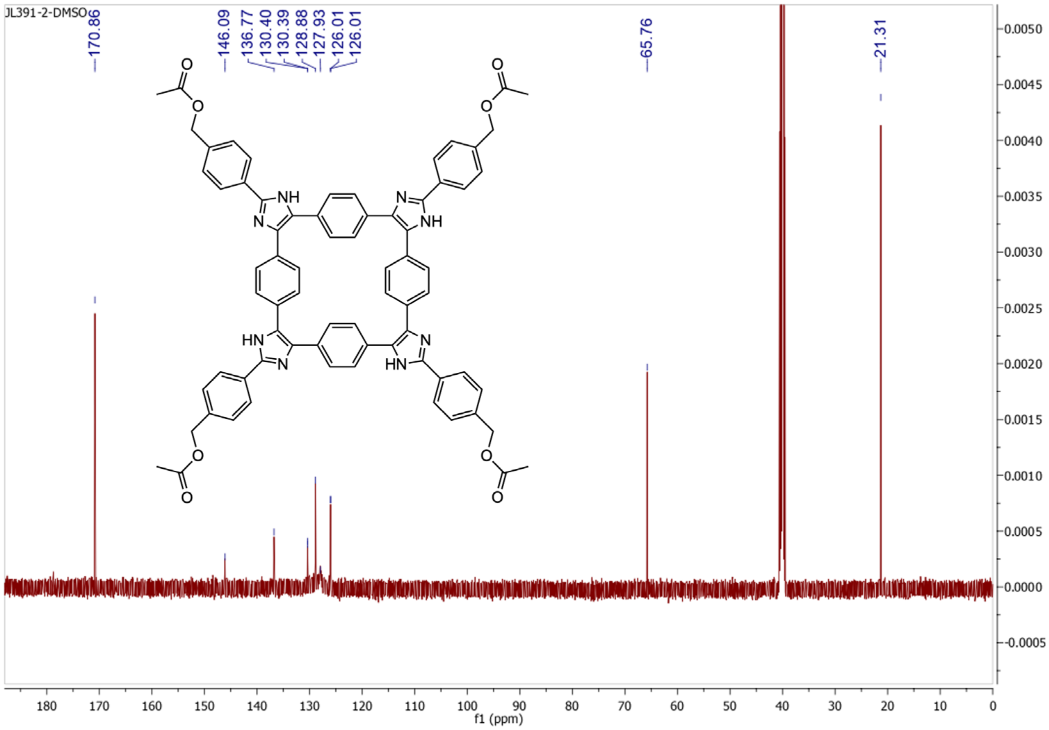

Figure S18. ^{13}C NMR spectrum of compound **3e** (126 MHz, DMSO-d_6, 25 °C).

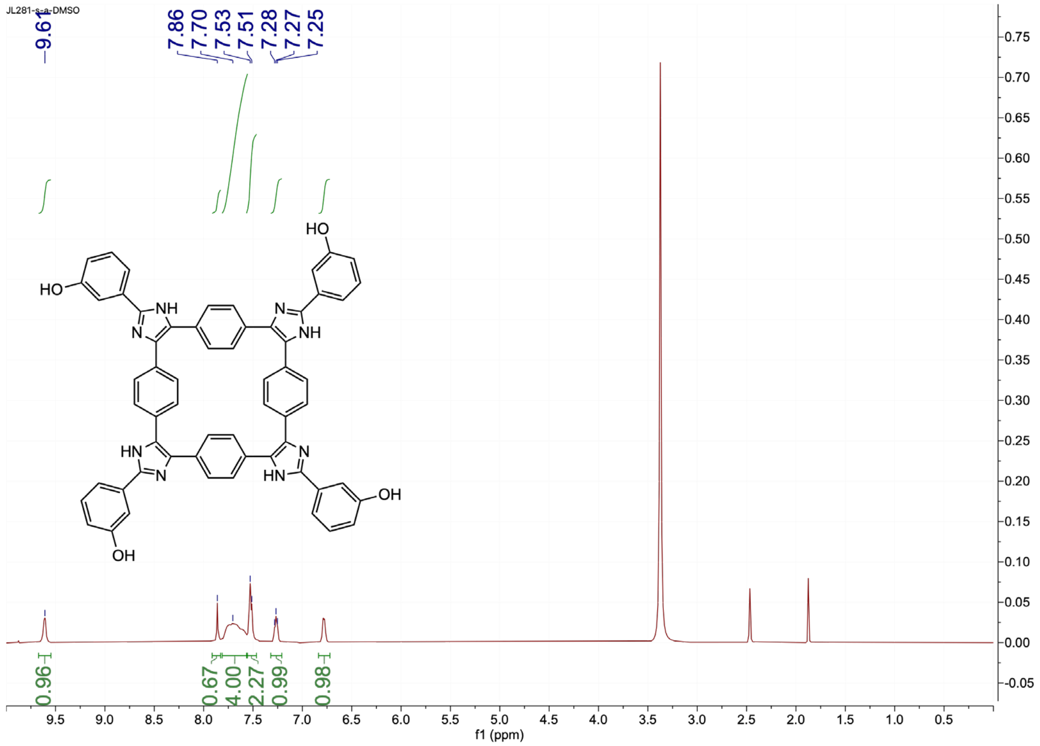

Figure S19. ^{1}H NMR spectrum of compound **3f** (500 MHz, DMSO-d_6, 25 °C).

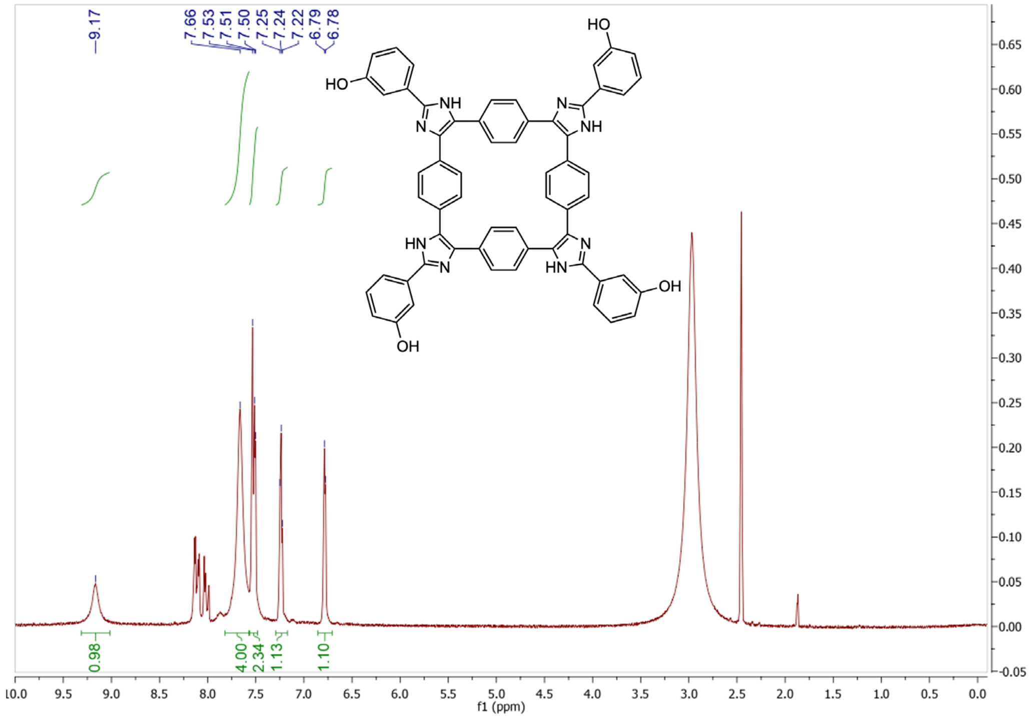

Figure S20. ^{1}H NMR spectrum of compound **3f** (600 MHz, DMSO-d_6, 100 °C).

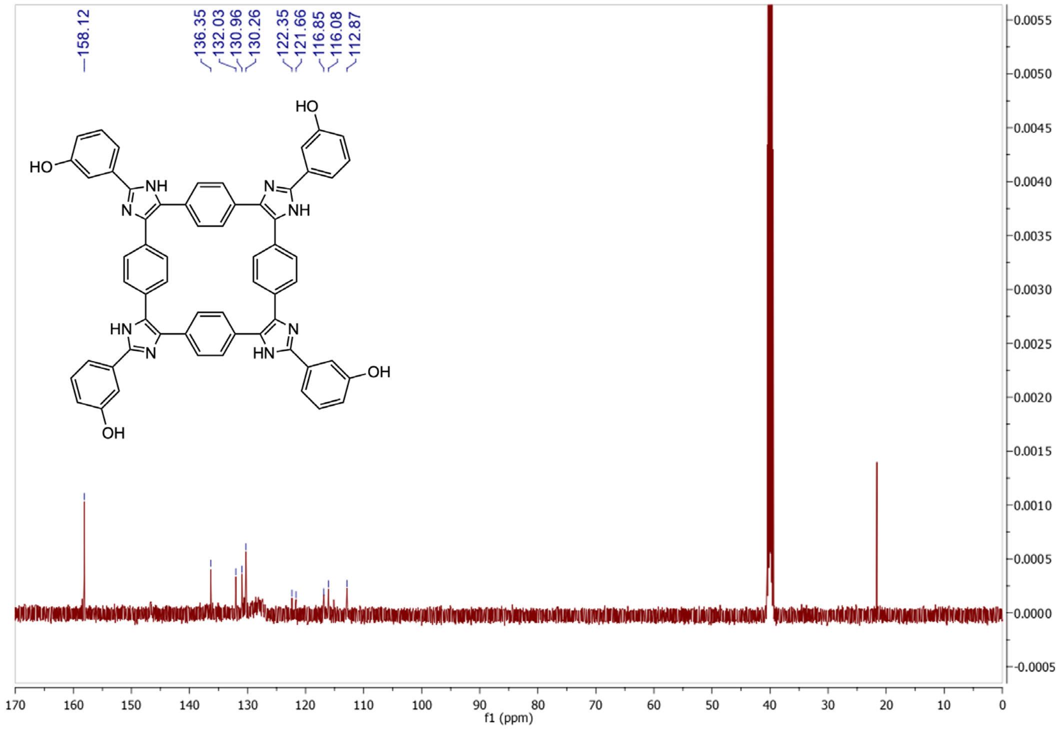

Figure S21. ^{13}C NMR spectrum of compound **3f** (126 MHz, DMSO-d_6, 25 °C).

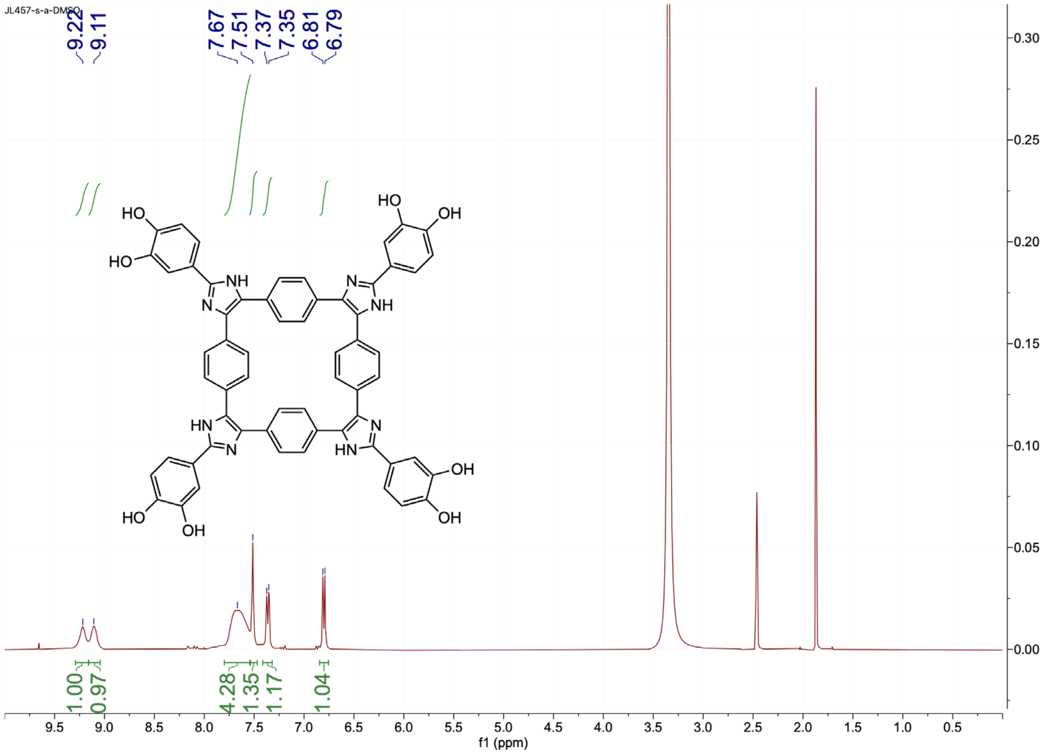

Figure S22. ^{1}H NMR spectrum of compound **3g** (400 MHz, DMSO-d_6, 25 °C).

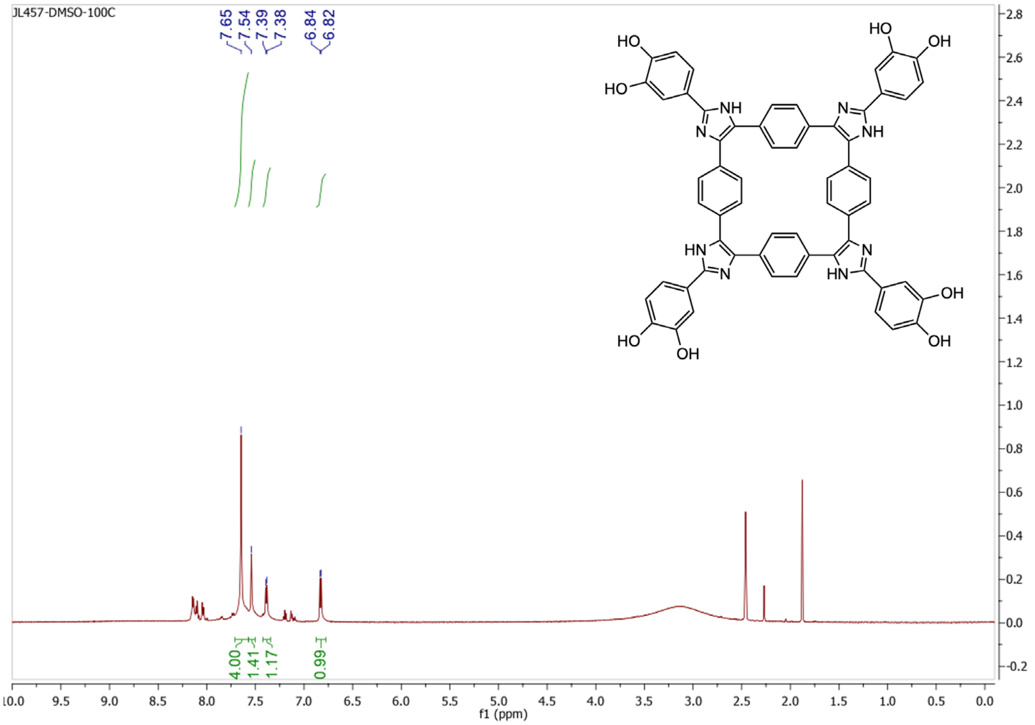

Figure S23. ^{1}H NMR spectrum of compound **3g** (600 MHz, DMSO-d_6, 100 °C).

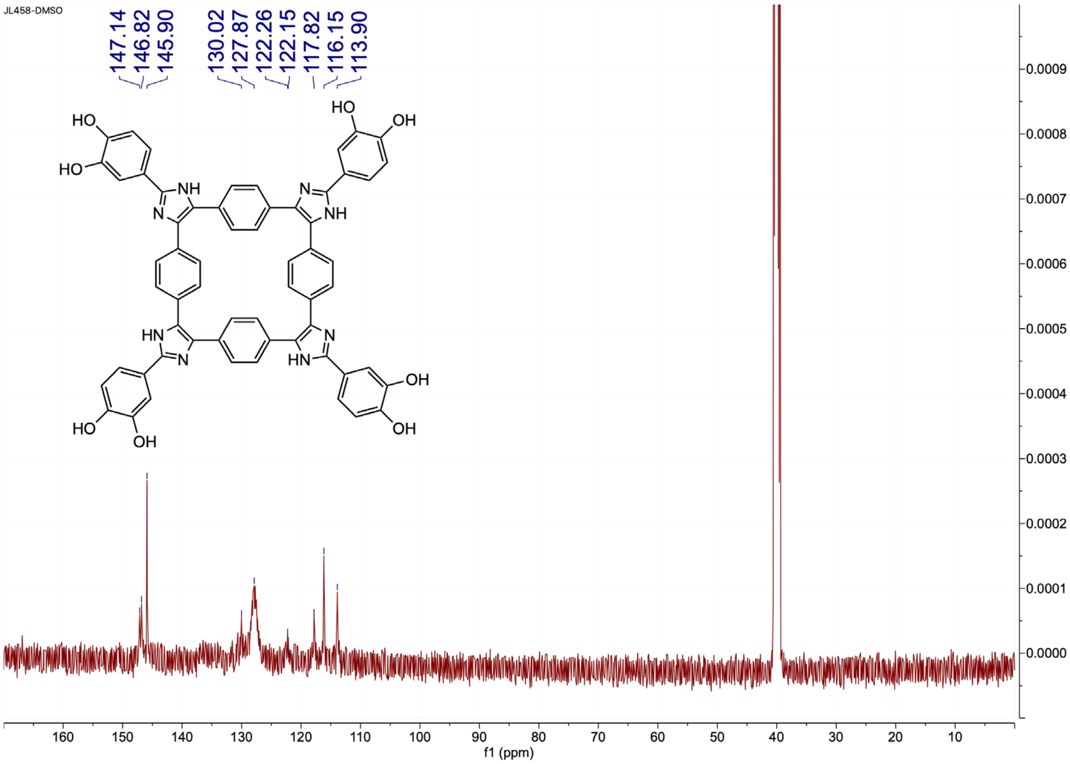

Figure S24. ^{13}C NMR spectrum of compound **3g** (126 MHz, DMSO-d_6, 25 °C).

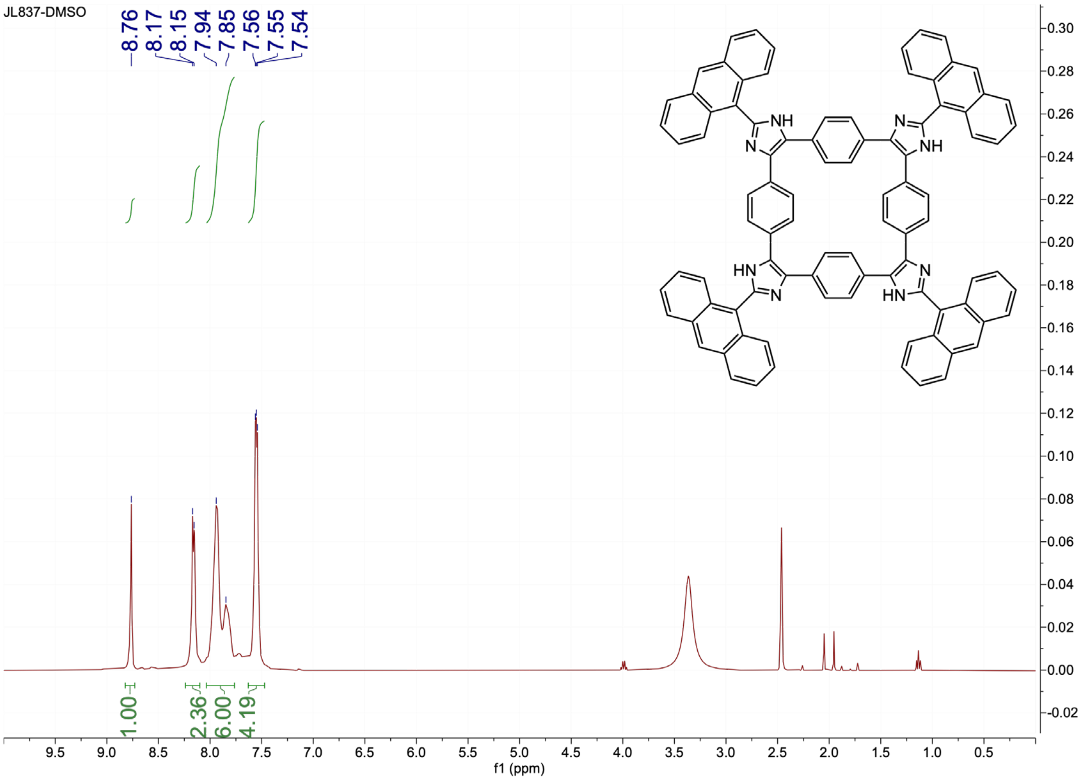

Figure S25. ^{1}H NMR spectrum of compound **3h** (500 MHz, DMSO-d_6, 25 °C).

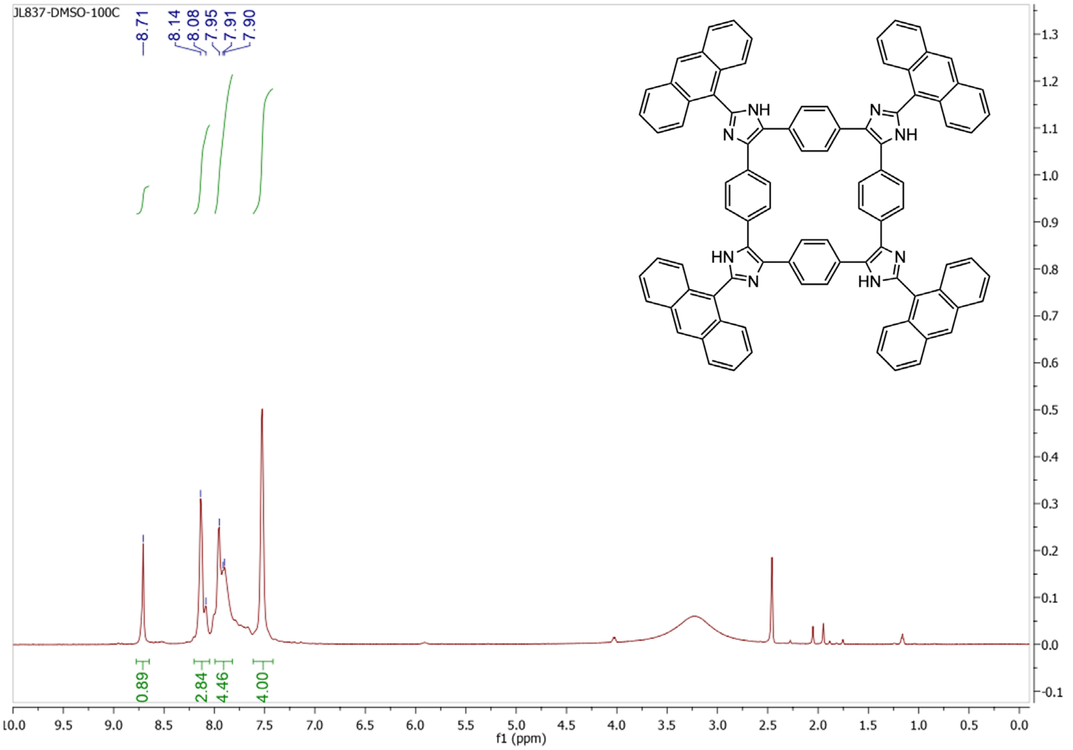

Figure S26. ^{1}H NMR spectrum of compound **3h** (600 MHz, DMSO-d_6, 100 °C).

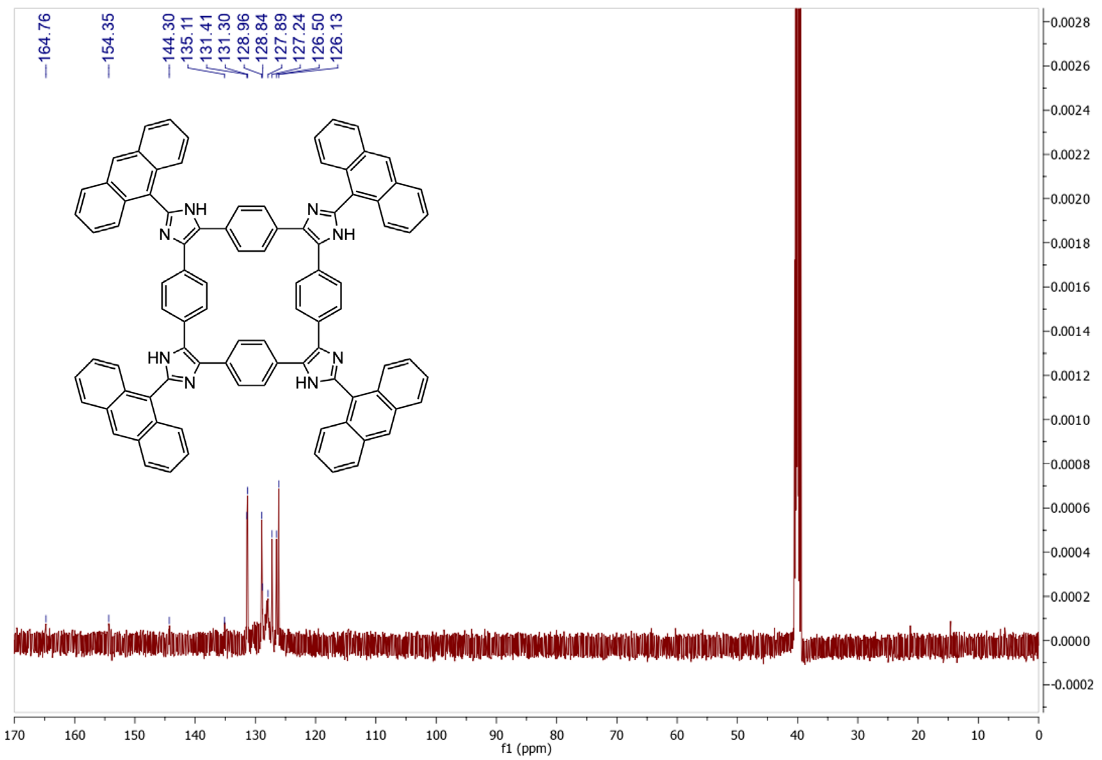

Figure S27. ^{13}C NMR spectrum of compound **3h** (126 MHz, DMSO-d_6, 25 °C).

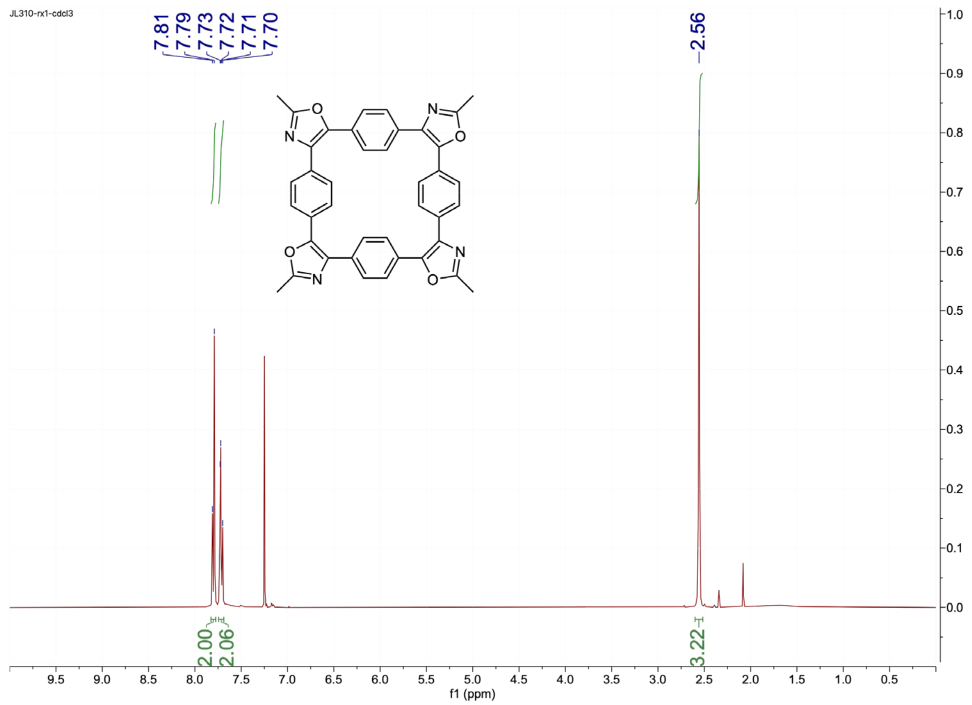

Figure S28. ^{1}H NMR spectrum of compound **5a** (400 MHz, $CDCl_3$, 25 °C).

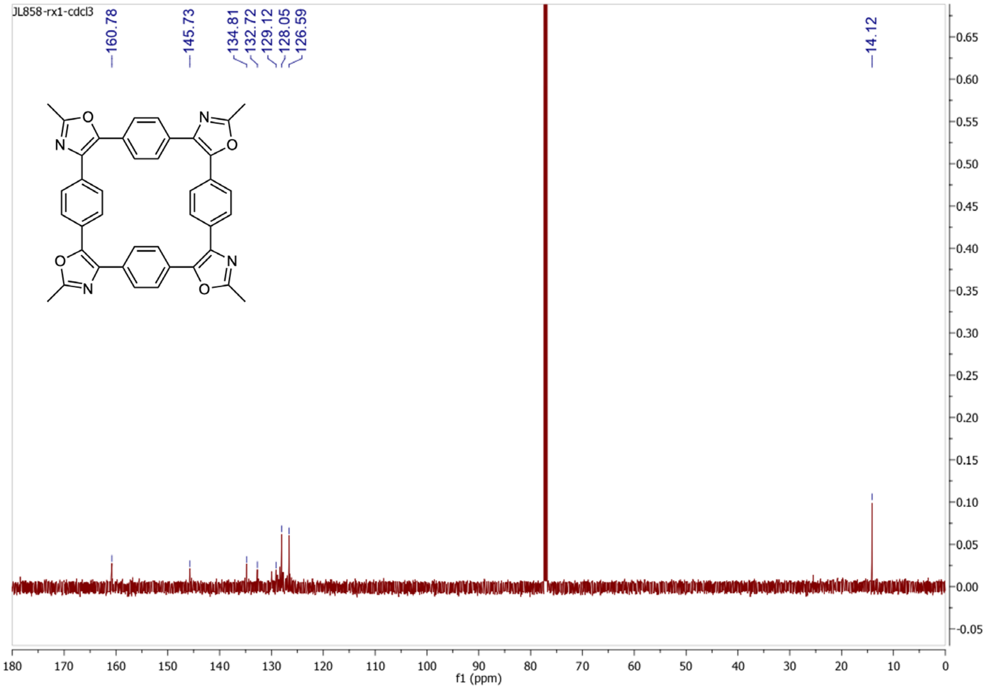

Figure S29. ^{13}C NMR spectrum of compound **5a** (151 MHz, $CDCl_3$, 25 °C).

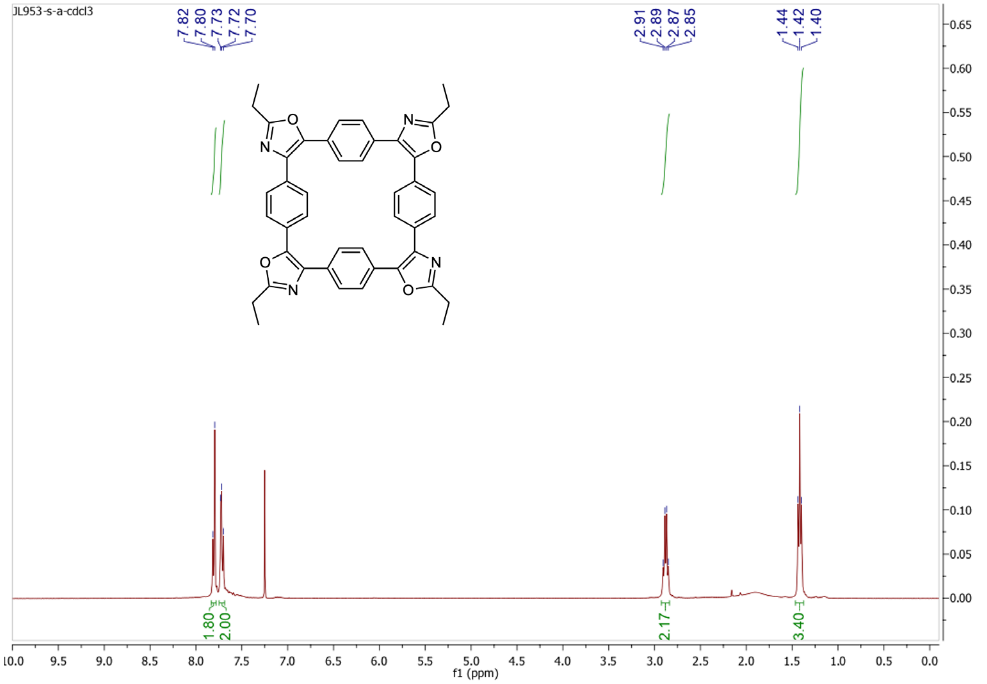

Figure S30. ^{1}H NMR spectrum of compound **5b** (400 MHz, $CDCl_3$, 25 °C).

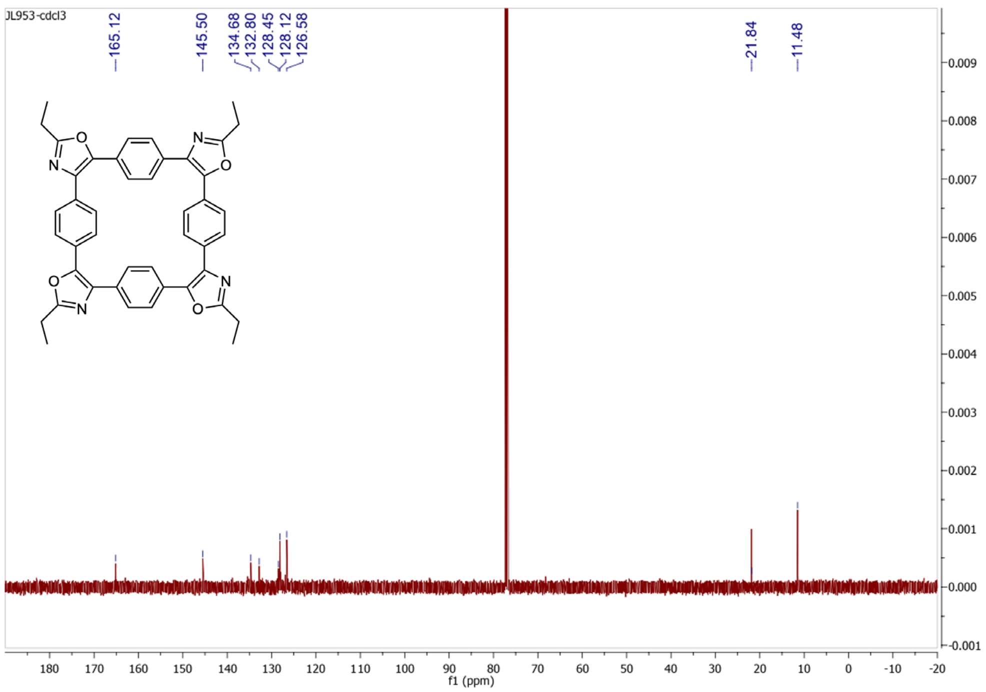

Figure S31. ^{13}C NMR spectrum of compound **5b** (126 MHz, $CDCl_3$, 25 °C).

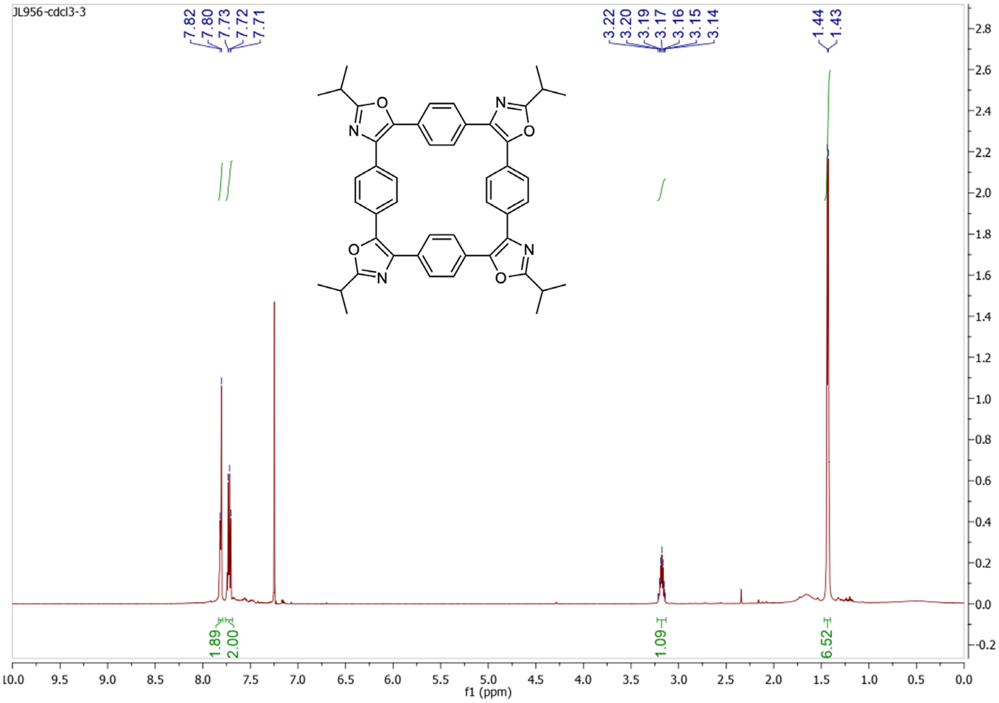

Figure S32. ^{1}H NMR spectrum of compound **5c** (600 MHz, $CDCl_3$, 25 °C).

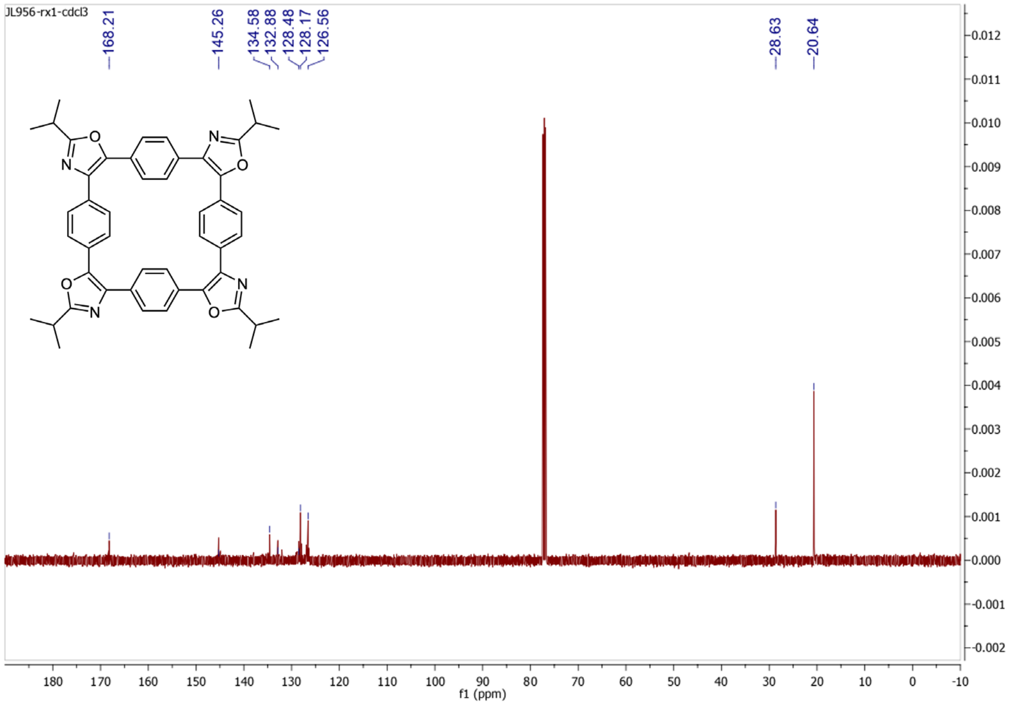

Figure S33. ^{13}C NMR spectrum of compound **5c** (126 MHz, $CDCl_3$, 25 °C).

Single Crystal Structures and Analyses

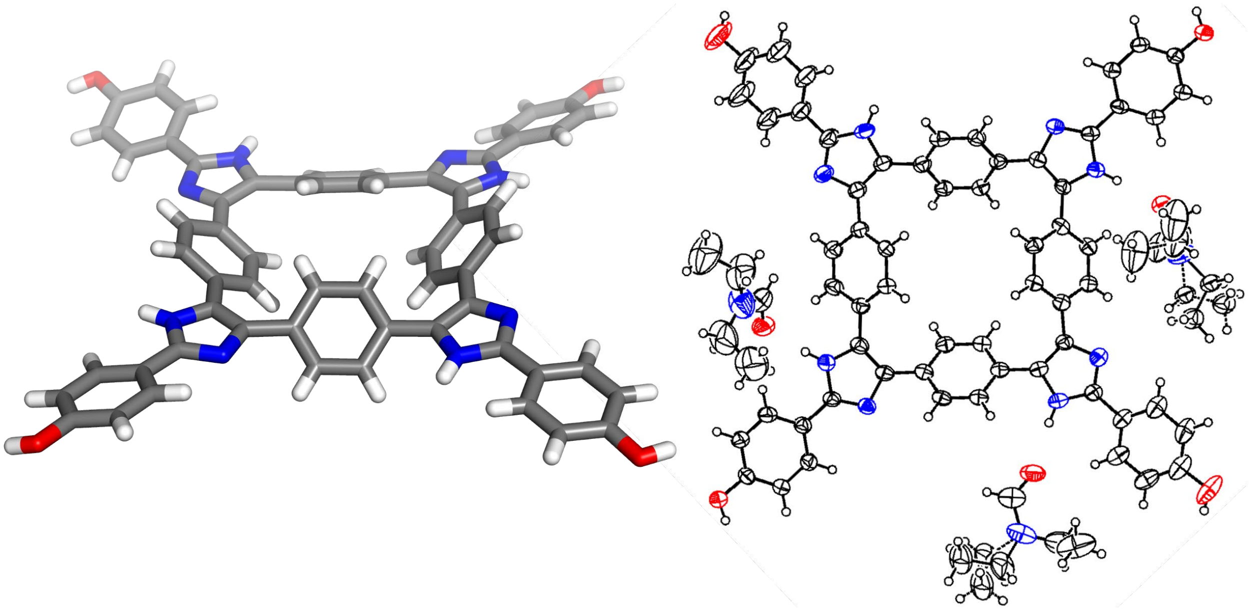

Figure S34. X-ray crystal structure (left) and ORTEP plot of the crystal structure of **3b** (right).

Table S1. Crystal data and structure refinement for compound **3b.**

Identification code	JL641_XW3084
Empirical formula	$C_{79}H_{79.8}N_{11}O_9$
Formula weight	1327.33
Temperature/K	100(2)
Crystal system	monoclinic
Space group	*P*2/*n*
a/Å	31.9415(8)
b/Å	9.5284(3)
c/Å	32.2718(9)
α/°	90
β/°	117.757(2)
γ/°	90
Volume/Å^3	8691.8(4)
Z	4
ρ_{calc} g/cm^3	1.014
μ/mm^{-1}	0.543
F(000)	2811.0

Crystal size/mm^3	0.237 × 0.124 × 0.01
Radiation	CuKα (λ = 1.54178)
2Θ range for data collection/°	5.326 to 136.806
Index ranges	$-33 \leq h \leq 38$, $-10 \leq k \leq 11$, $-38 \leq l \leq 38$
Reflections collected	74150
Independent reflections	15437 [R_{int} = 0.1168, R_{sigma} = 0.0972]
Data/restraints/parameters	15437/262/951
Goodness-of-fit on F^2	1.129
Final R indexes [$I \geq 2\sigma(I)$]	R_1 = 0.0899, wR_2 = 0.1930
Final R indexes [all data]	R_1 = 0.1737, wR_2 = 0.2290
Largest diff. peak/hole / e Å^{-3}	0.51/−0.32

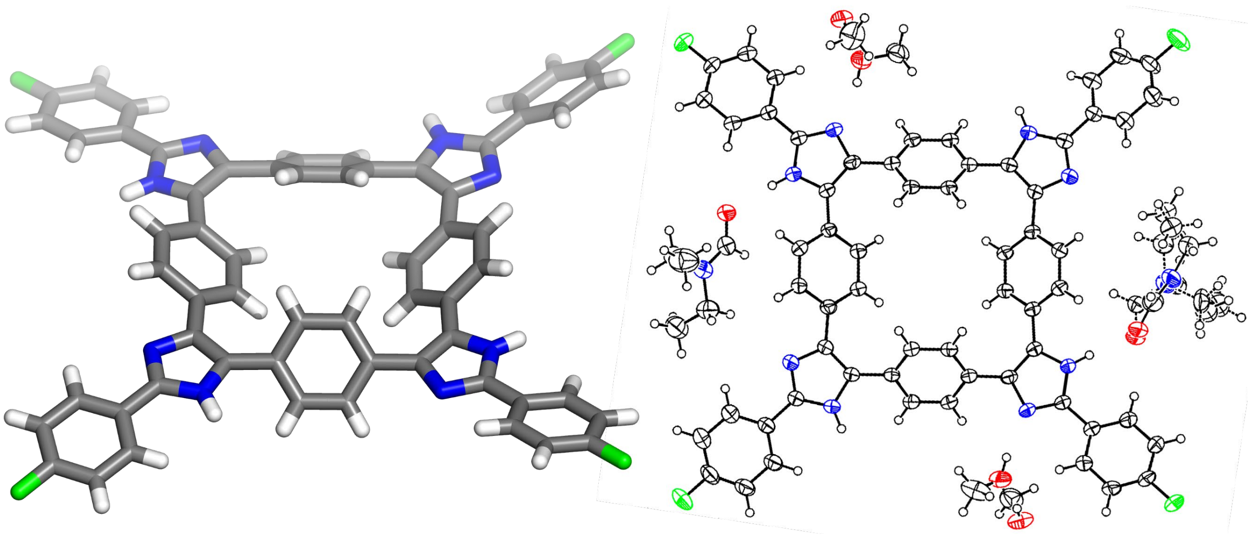

Figure S35. X-ray crystal structure (left) and ORTEP plot of the crystal structure of **3c** (right).

Table S2. Crystal data and structure refinement for compound **3c**.

Identification code	JL642_XW604
Empirical formula	$C_{74}H_{74}F_4N_{10}O_6$
Formula weight	1275.43
Temperature/K	123(2)
Crystal system	triclinic
Space group	$P\bar{1}$
a/Å	10.4421(3)
b/Å	15.6560(4)
c/Å	21.4219(5)
α/°	74.348(2)

β/°	75.957(2)
γ/°	78.119(2)
Volume/Å^3	3234.27(15)
Z	2
ρ_{calc} g/cm^3	1.310
μ/mm^{-1}	0.757
F(000)	1344.0
Crystal size/mm^3	0.38 × 0.03 × 0.01
Radiation	CuKα (λ = 1.54178)
2Θ range for data collection/°	4.37 to 134.992
Index ranges	$-12 \leq h \leq 12$, $-18 \leq k \leq 17$, $-25 \leq l \leq 25$
Reflections collected	52586
Independent reflections	11223 [R_{int} = 0.0604, R_{sigma} = 0.0714]
Data/restraints/parameters	11223/12/895
Goodness-of-fit on F^2	1.045
Final R indexes [$I \geq 2\sigma(I)$]	R_1 = 0.0562, wR_2 = 0.1494
Final R indexes [all data]	R_1 = 0.0875, wR_2 = 0.1714
Largest diff. peak/hole / e Å^{-3}	0.47/−0.26

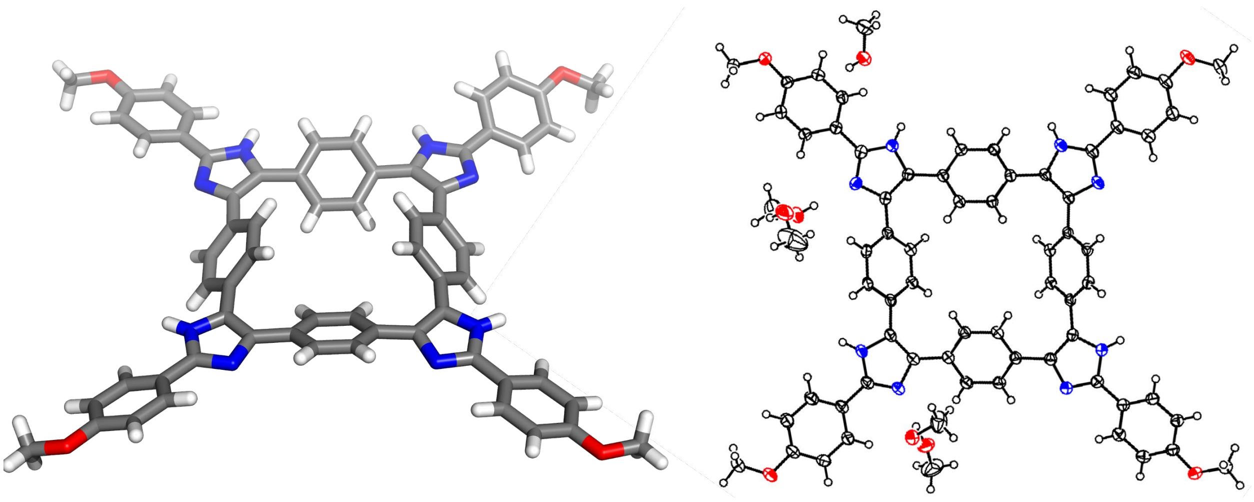

Figure S36. X-ray crystal structure (left) and ORTEP plot of the crystal structure of **3d** (right).

Table S3. Crystal data and structure refinement for compound **3d**.

Identification code	JL856_XW3080_sq
Empirical formula	$C_{69}H_{68}N_8O_9$
Formula weight	1153.31
Temperature/K	100(2)

Crystal system	monoclinic
Space group	$P2_1/n$
a/Å	14.7923(4)
b/Å	22.1907(6)
c/Å	20.6091(5)
α/°	90
β/°	95.371(2)
γ/°	90
Volume/Å^3	6735.3(3)
Z	4
ρ_{calc} g/cm^3	1.137
μ/mm^{-1}	0.616
F(000)	2440.0
Crystal size/mm^3	0.245 × 0.094 × 0.061
Radiation	CuKα (λ = 1.54178)
2Θ range for data collection/°	5.866 to 140.202
Index ranges	$-18 \leq h \leq 17$, $-27 \leq k \leq 27$, $-25 \leq l \leq 25$
Reflections collected	110850
Independent reflections	12761 [R_{int} = 0.0364, R_{sigma} = 0.0232]
Data/restraints/parameters	12761/6/791
Goodness-of-fit on F^2	1.041
Final R indexes [$I \geq 2\sigma(I)$]	R_1 = 0.0539, wR_2 = 0.1507
Final R indexes [all data]	R_1 = 0.0595, wR_2 = 0.1566
Largest diff. peak/hole / e Å^{-3}	0.68/−0.36

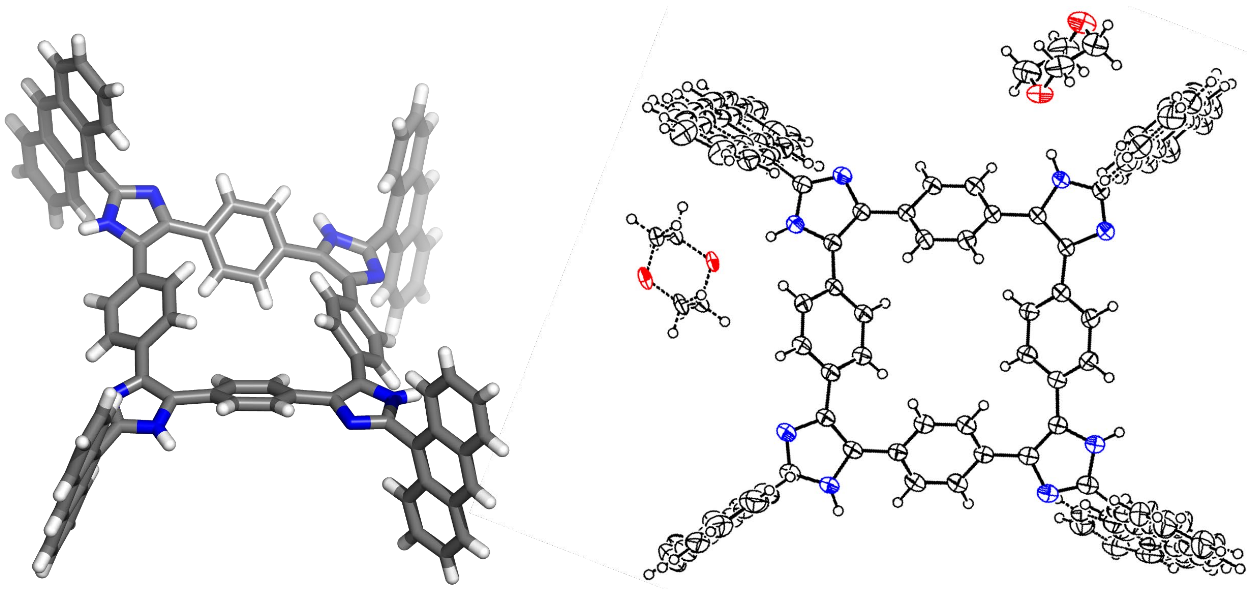

Figure S37. X-ray crystal structure (left) and ORTEP plot of the crystal structure of **3h** (right).

Table S4. Crystal data and structure refinement for compound **3h**.

Identification code	JL752-3_XW669_sq
Empirical formula	$C_{97.5}H_{67}N_8O_{2.75}$
Formula weight	1394.59
Temperature/K	123(2)
Crystal system	triclinic
Space group	$P\bar{1}$
a/Å	15.6863(4)
b/Å	19.4786(5)
c/Å	21.1524(4)
α/°	62.9460(10)
β/°	78.805(2)
γ/°	86.651(2)
Volume/Å^3	5643.0(2)
Z	2
ρ_{calc} g/cm^3	0.821
μ/mm^{-1}	0.391
F(000)	1460.0
Crystal size/mm^3	0.21 × 0.18 × 0.17
Radiation	CuKα (λ = 1.54178)
2Θ range for data collection/°	5.096 to 136.584
Index ranges	$-18 \le h \le 18, -23 \le k \le 23, -25 \le l \le 24$
Reflections collected	68280

Independent reflections	19836 [R_{int} = 0.0307, R_{sigma} = 0.0409]
Data/restraints/parameters	19836/15530/1330
Goodness-of-fit on F^2	1.063
Final R indexes [$I \geq 2\sigma(I)$]	R_1 = 0.0711, wR_2 = 0.2284
Final R indexes [all data]	R_1 = 0.0851, wR_2 = 0.2452
Largest diff. peak/hole / e Å^{-3}	0.53/−0.22

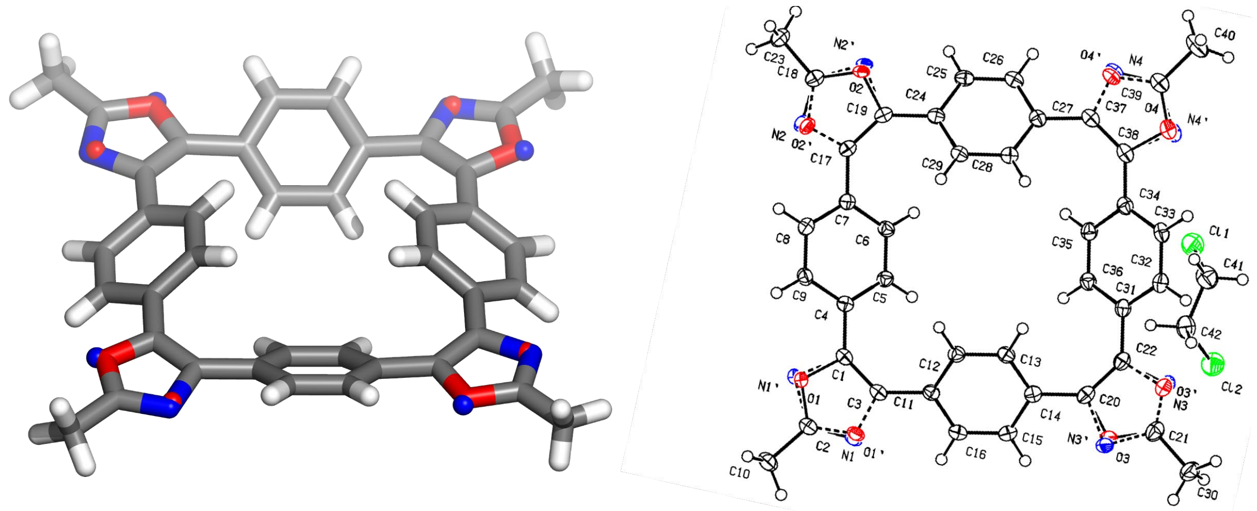

Figure S38. X-ray crystal structure (left) and ORTEP plot of the crystal structure of **5a** (right).

Table S5. Crystal data and structure refinement for compound **5a**.

Identification code	JL310-14_XW511
Empirical formula	$C_{42}H_{32}Cl_2N_4O_4$
Formula weight	727.61
Temperature/K	123(2)
Crystal system	monoclinic
Space group	$P2_1/c$
a/Å	16.7306(2)
b/Å	8.77900(10)
c/Å	24.8572(3)
α/°	90
β/°	109.0600(10)
γ/°	90
Volume/Å^3	3450.82(7)
Z	4
ρ_{calc} g/cm^3	1.401

μ/mm^{-1}	2.108
F(000)	1512.0
Crystal size/mm^3	0.45 × 0.04 × 0.03
Radiation	CuKα (λ = 1.54178)
2Θ range for data collection/°	5.588 to 132.942
Index ranges	$-18 \leq h \leq 19$, $-9 \leq k \leq 9$, $-28 \leq l \leq 29$
Reflections collected	34272
Independent reflections	5964 [R_{int} = 0.0280, R_{sigma} = 0.0212]
Data/restraints/parameters	5964/563/498
Goodness-of-fit on F^2	1.080
Final R indexes [$I \geq 2\sigma (I)$]	R_1 = 0.0399, wR_2 = 0.1171
Final R indexes [all data]	R_1 = 0.0429, wR_2 = 0.1197
Largest diff. peak/hole / e Å^{-3}	0.29/−0.47

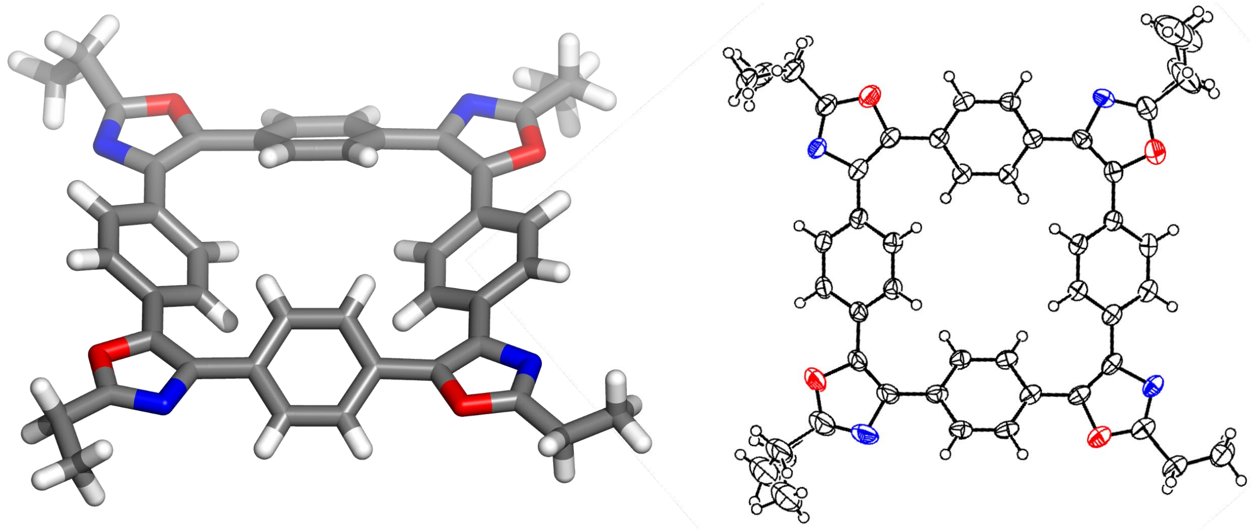

Figure S39. X-ray crystal structure (left) and ORTEP plot of the crystal structure of **5b** (right).

Table S6. Crystal data and structure refinement for compound **5b**.

Identification code	JL953-14_XW3202B_sq
Empirical formula	$C_{44}H_{36}N_4O_4$
Formula weight	684.77
Temperature/K	100(2)
Crystal system	monoclinic

Space group	$P2/c$
a/Å	13.9568(3)
b/Å	8.8485(2)
c/Å	31.0653(6)
α/°	90
β/°	102.3130(10)
γ/°	90
Volume/Å^3	3748.21(14)
Z	4
ρ_{calc} g/cm^3	1.213
μ/mm^{-1}	0.628
F(000)	1440.0
Crystal size/mm^3	0.672 × 0.101 × 0.083
Radiation	CuKα (λ = 1.54178)
2Θ range for data collection/°	5.824 to 149.196
Index ranges	$-17 \le h \le 16, -11 \le k \le 10, -38 \le l \le 38$
Reflections collected	46685
Independent reflections	7591 [R_{int} = 0.0648, R_{sigma} = 0.0448]
Data/restraints/parameters	7591/562/492
Goodness-of-fit on F^2	1.081
Final R indexes [$I \ge 2\sigma(I)$]	R_1 = 0.0816, wR_2 = 0.2083
Final R indexes [all data]	R_1 = 0.0860, wR_2 = 0.2116
Largest diff. peak/hole / e Å^{-3}	0.89/–0.54

UV-Vis Spectroscopic Titrations

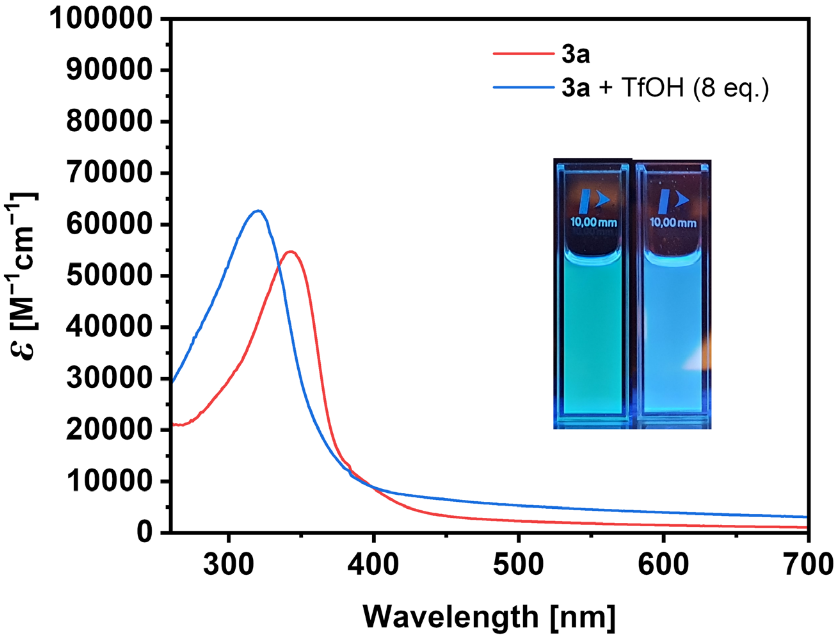

Figure S40. UV/Vis absorption spectrum of an 8.9×10^{-6} M solution of **3a** in THF before (red curve) and after (blue curve) the addition of 8 eq. of trifluoromethanesulfonic acid (TfOH). Insert shows photographs of the fluorescence emission of **3a** under a UV lamp with λ_{exc} of 254 nm (left: before TfOH addition, right: after TfOH addition).

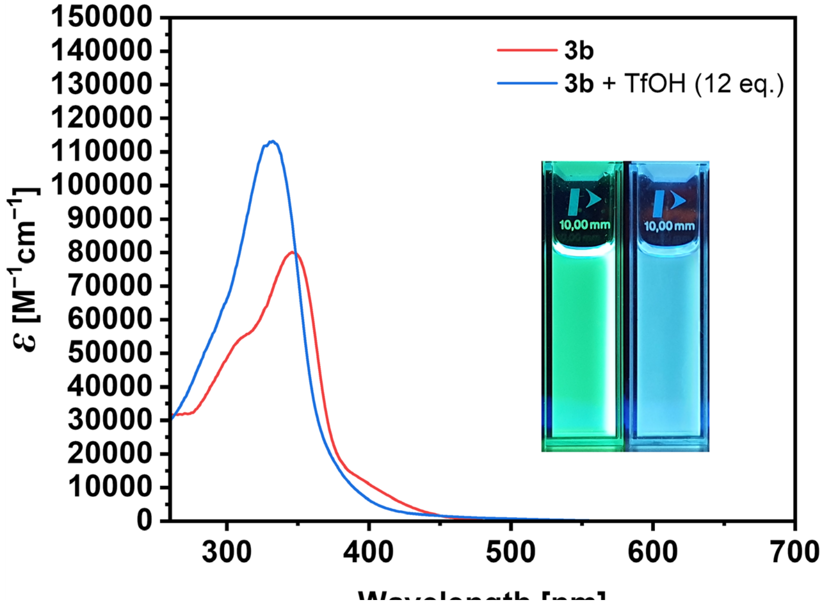

Figure S41. UV/Vis absorption spectrum of an 8.3×10^{-6} M solution of **3b** in THF before (red curve) and after (blue curve) the addition of 12 eq. of TfOH. Insert shows photographs of the fluorescence emission of **3b** under a UV lamp with λ_{exc} of 254 nm (left: before TfOH addition, right: after TfOH addition).

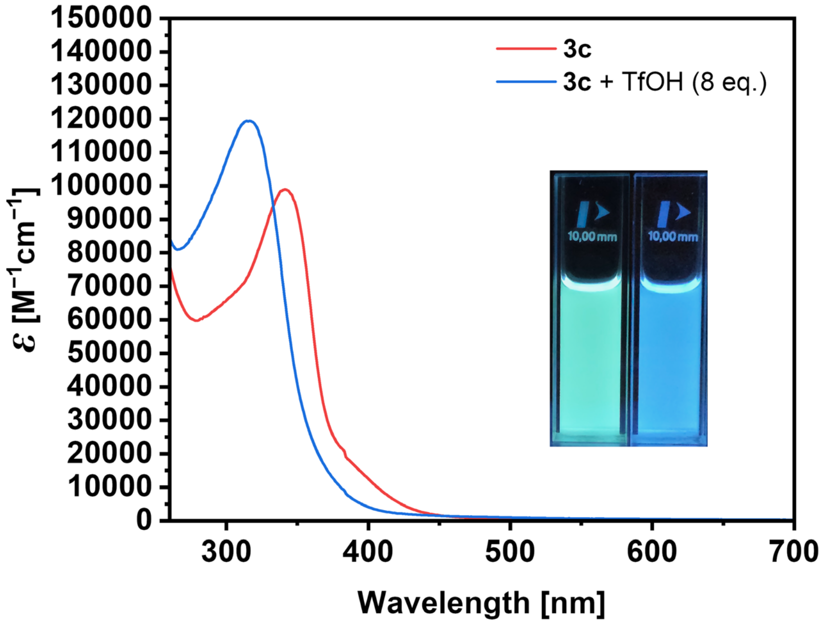

Figure S42. UV/Vis absorption spectrum of a 7.2×10^{-6} M solution of **3c** in THF before (red curve) and after (blue curve) the addition of 8 eq. of TfOH. Insert shows photographs of the fluorescence emission of **3c** under a UV lamp with λ_{exc} of 254 nm (left: before TfOH addition, right: after TfOH addition).

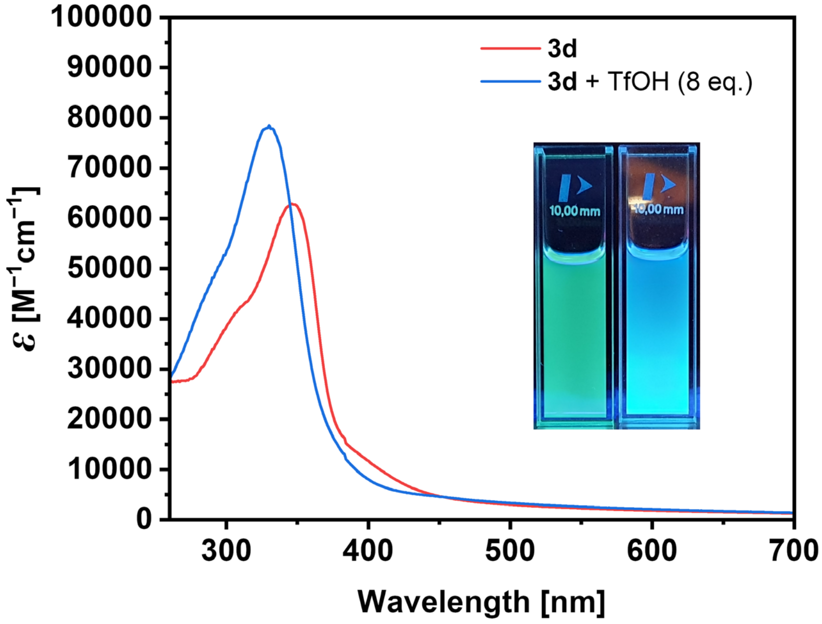

Figure S43. UV/Vis absorption spectrum of an 8.3×10^{-6} M solution of **3d** in THF before (red curve) and after (blue curve) the addition of 8 eq. of TfOH. Insert shows photographs of the fluorescence emission of **3d** under a UV lamp with λ_{exc} of 254 nm (left: before TfOH addition, right: after TfOH addition).

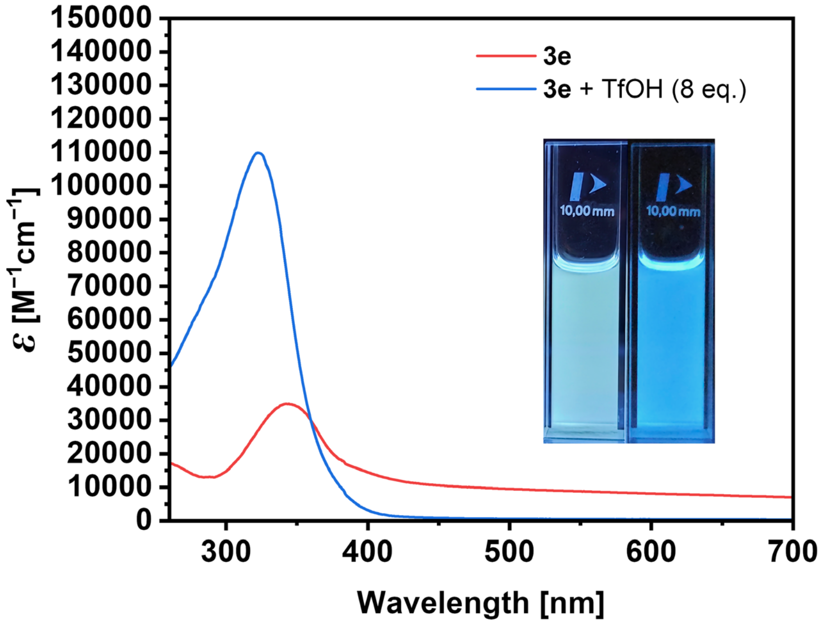

Figure S44. UV/Vis absorption spectrum of a 6.9×10^{-6} M solution of **3e** in THF before (red curve) and after (blue curve) the addition of 8 eq. of TfOH. Insert shows photographs of the fluorescence emission of **3e** under a UV lamp with λ_{exc} of 254 nm (left: before TfOH addition, right: after TfOH addition).

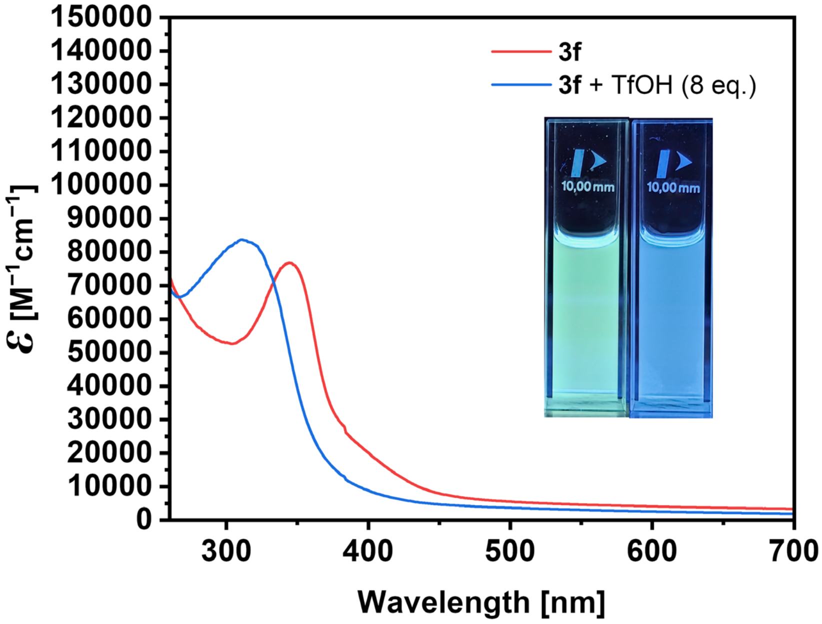

Figure S45. UV/Vis absorption spectrum of a 9.4×10^{-6} M solution of **3f** in THF before (red curve) and after (blue curve) the addition of 8 eq. of TfOH. Insert shows photographs of the fluorescence emission of **3f** under a UV lamp with λ_{exc} of 254 nm (left: before TfOH addition, right: after TfOH addition).

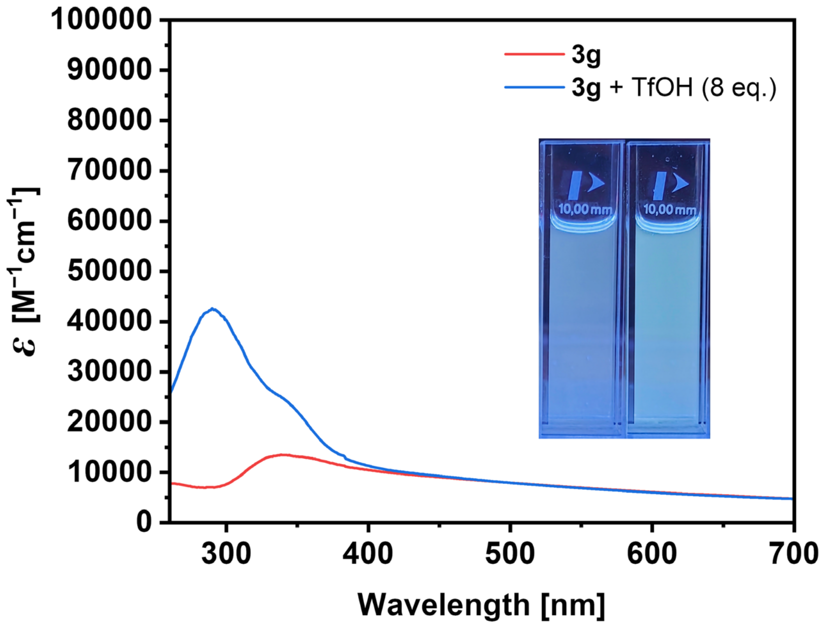

Figure S46. UV/Vis absorption spectrum of a 1.7×10^{-5} M solution of **3g** in THF before (red curve) and after (blue curve) the addition of 8 eq. of TfOH. Insert shows photographs of the fluorescence emission of **3g** under a UV lamp with λ_{exc} of 254 nm (left: before TfOH addition, right: after TfOH addition).

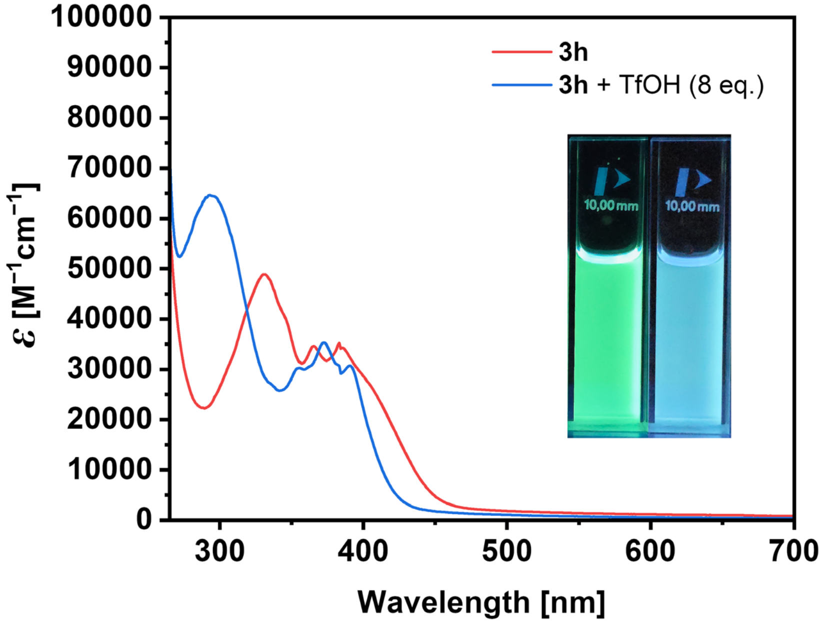

Figure S47. UV/Vis absorption spectrum of an 8.5×10^{-6} M solution of **3h** in THF before (red curve) and after (blue curve) the addition of 8 eq. of TfOH. Insert shows photographs of the fluorescence emission of **3h** under a UV lamp with λ_{exc} of 254 nm (left: before TfOH addition, right: after TfOH addition).

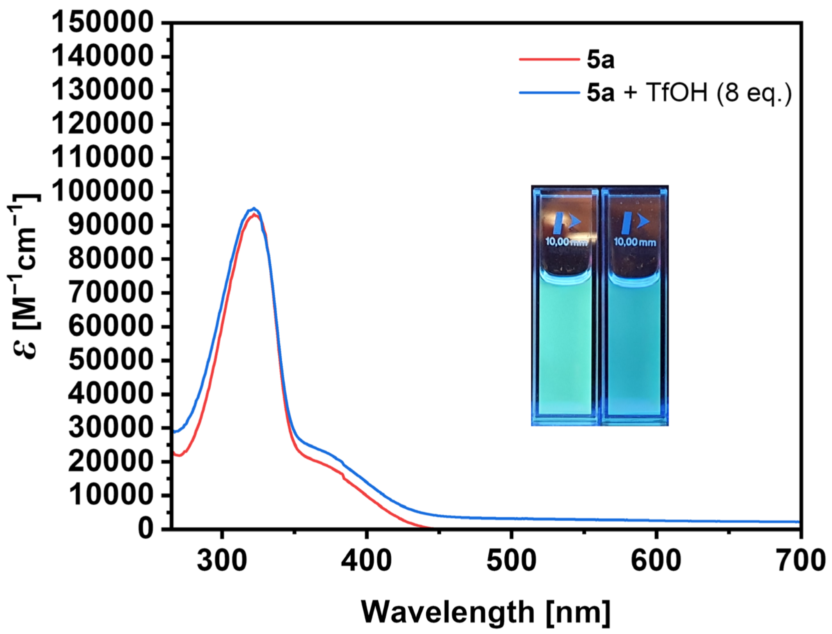

Figure S48. UV/Vis absorption spectrum of a 7.8×10^{-6} M solution of **5a** in THF before (red curve) and after (blue curve) the addition of 8 eq. of TfOH. Insert shows photographs of the fluorescence emission of **5a** under a UV lamp with λ_{exc} of 254 nm (left: before TfOH addition, right: after TfOH addition).

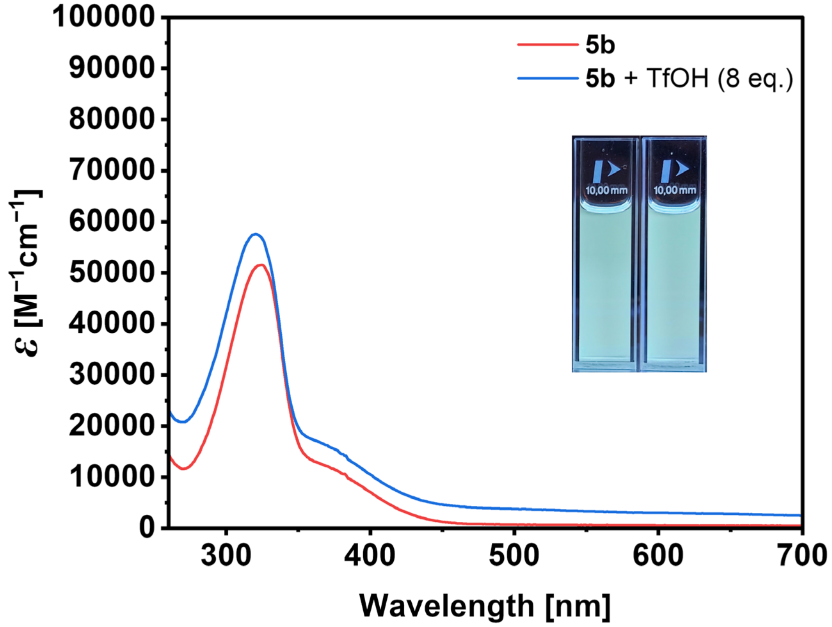

Figure S49. UV/Vis absorption spectrum of a 5.8×10^{-6} M solution of **5b** in THF before (red curve) and after (blue curve) the addition of 8 eq. of TfOH. Insert shows photographs of the fluorescence emission of **5b** under a UV lamp with λ_{exc} of 254 nm (left: before TfOH addition, right: after TfOH addition).

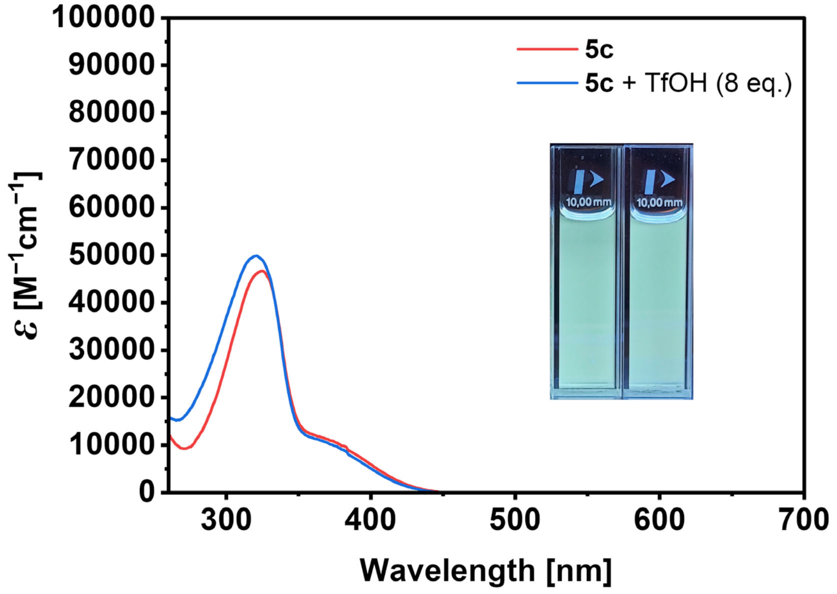

Figure S50. UV/Vis absorption spectrum of a 5.7×10^{-6} M solution of **5c** in THF before (red curve) and after (blue curve) the addition of 8 eq. of TfOH. Insert shows photographs of the fluorescence emission of **5c** under a UV lamp with λ_{exc} of 254 nm (left: before TfOH addition, right: after TfOH addition).

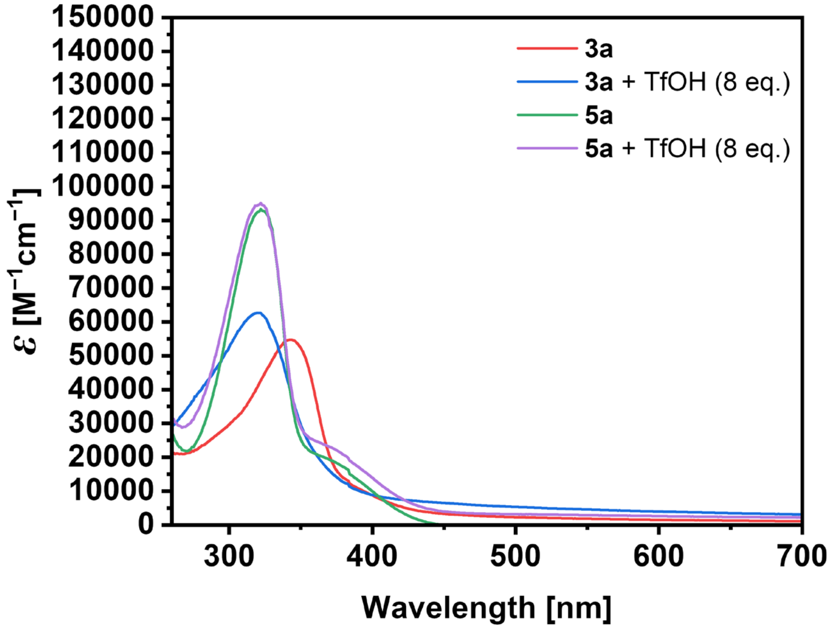

Figure S51. Comparison of optical responses of **3a** (8.9×10^{-6} M solution in THF; before—red curve, after—blue curve) and **5a** (7.8×10^{-6} M solution in THF; before—green cruve, after—magenta curve) to TfOH addition.

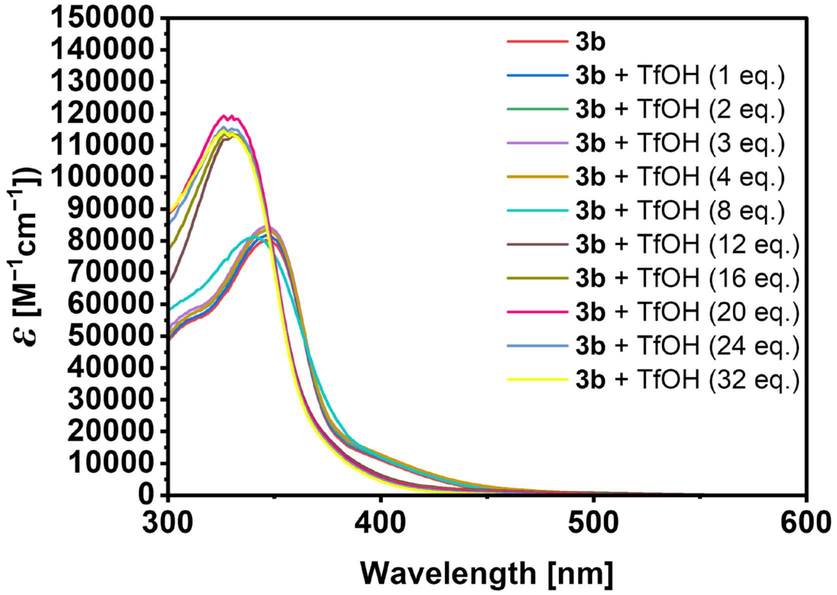

Figure S52. Stacked UV/Vis absorption spectra of the titration of an 8.3×10^{-6} M solution of **3b** in THF with TfOH.

Table S7. Absorption maxima for compounds **3a**–**h** and **5a**–**c** before and after addition of TfOH.

	Original<br>λ [nm], max ε [M^{-1} cm^{-1}]	After addition of TfOH (8 eq.)<br>λ [nm], max ε [M^{-1} cm^{-1}]
**3a**	347, 53876	323, 62157
**3b**	349, 77397	337, 110096
**3c**	332, 96431	321, 117694
**3d**	446, 60084	334, 76939
**3e**	349, 34319	328, 106362
**3f**	350, 75064	317, 82809
**3g**	339, 13465	296, 41324
**3h**	336, 46894	300, 63106
**5a**	327, 91526	326, 93372
**5b**	331, 50914	324, 57052
**5c**	329, 46140	325, 48947

Computational Analysis of Protonation

We carried out a series of computationally efficient simulations to estimate the thermodynamic feasibility of stepwise protonation. Gibbs free energies (G, in kcal/mol) for the neutral form and for the mono-, di-, tri-, and tetra-protonated species were computed at the wB97X-D4/def2-TZVPP level of theory with CPCM(MeCN) solvation at 298 K. The resulting values of ΔG_{prot} for each step of protonation are as follows: −277.47 kcal/mol (**3c**→**3c**·H^+), −275.35 kcal/mol (**3c**·H^+→**3c**·$2H^+$), −273.99 kcal/mol (**3c**·$2H^{2+}$→**3c**·$3H^{3+}$), and −272.34 kcal/mol (**3c**·$3H^{3+}$→**3c**·$4H^{4+}$). The standard-state free energy of a proton in acetonitrile (MeCN), was assumed to be −254.20 kcal/mol.[4] Compared to this reference value, our stepwise protonation free energies (ΔG_{prot}) fall within the range of moderately to slightly exergonic values, from –23.27 kcal/mol for the first protonation to –18.14 kcal/mol for the fourth protonation. This indicates that all four nitrogen atoms can be protonated under the conditions of TfOH treatment, and that tetraprotonation remains thermodynamically feasible. No discontinuities or energetic barriers were observed between protonation steps, and the correlation between total Gibbs free energy and number of protons is perfectly linear (R^2 = 1.0000), suggesting no electronic or steric resistance to further protonation. Moreover, BDF analysis reveals that the global effectiveness of π-delocalization gradually decreases with each additional proton, from 56.36e (**3c**) to 54.64e (**3c**·$4H^{4+}$). This decline is also linear (R^2 = 0.9936), and as shown in the BDF maps (Figure S53), it is primarily caused by enhanced local charge resonance within the protonated five-membered rings, which weakens conjugation of the embedded π-bonds (i.e., the coherent π-delocalization gives way to resonance involving localized positive charges).

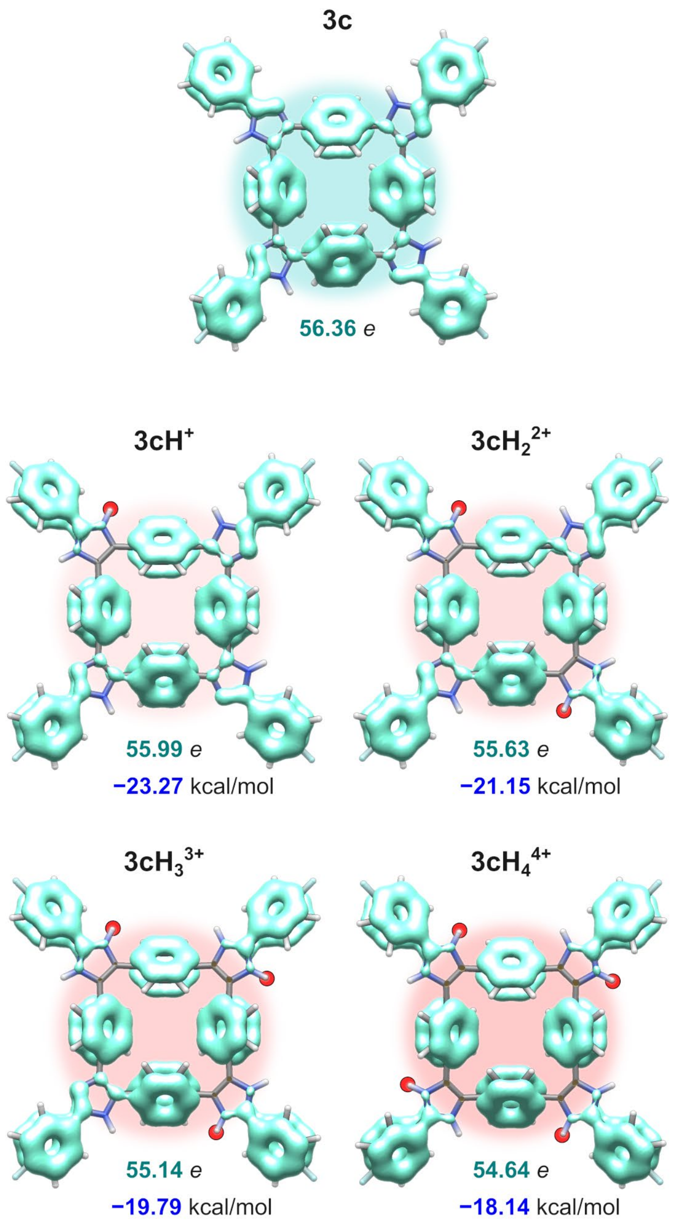

Figure S53. BDF maps (isovalue = 0.010) and total BDF-based populations of delocalized electrons (in teal) shown for the neutral form (**3c**) and its mono-, di-, tri-, and tetra-protonated analogues (**3c**·H^+ to **3c**·$4H^{4+}$). The associated Gibbs free energies of protonation (in kcal/mol, blue) were calculated relative to a standard-state proton in acetonitrile ($\Delta G°(H^+)$ = –254.20 kcal/mol; see: W. R. Fawcett, *Langmuir* **2008**, *24*, 9868, DOI: 10.1021/la7038976). The data demonstrates that all four nitrogen atoms can be protonated under TfOH treatment, as each step remains thermodynamically favorable. Simultaneously, the global efficiency of π-delocalization systematically decreases from 56.36e to 54.64e due to increasing charge resonance localized within the five-membered rings (the coherent π-bond conjugation gives way to the π-resonance involving positive charges). Geometry optimizations and thermochemical data were obtained at the wB97X-D4rev/def2-TZVPP level with CPCM(MeCN) solvation at 298 K using ORCA 6.1.0.

References

[1] Ji, Q.; Le, H. T. M.; Wang, X.; Chen, Y.-S.; Makarenko, T.; Jacobson, A. J.; Miljanić, O. Š. Cyclotetrabenzoin: Facile Synthesis of a Shape-Persistent Molecular Square and Its Assembly into Hydrogen-Bonded Nanotubes. *Chem. Eur. J.* **2015**, *21*, 17205–17209.

[2] McHale, C. M.; Stegemoller, C. R.; Hashim, M. I.; Wang, X.; Miljanić, O. Š. Porosity and Guest Inclusion in Cyclobenzoin Esters. *Cryst. Growth Des.* **2019**, *19*, 562–567.

[3] Alrayyani, M.; Miljanić, O. Š. Benzoins and Cyclobenzoins in Supramolecular and Polymer Chemistry. *Chem. Commun.* **2018**, *54*, 11989–11997.

[4] Fawcett, W. R. The Ionic Work Function and its Role in Estimating Absolute Electrode Potentials. *Langmuir* **2008**, *24*, 9868–9875.
